# Functional human IgA targets a conserved site on malaria sporozoites

**DOI:** 10.1126/scitranslmed.abg2344

**Published:** 2021-06-23

**Authors:** Joshua Tan, Hyeseon Cho, Tossapol Pholcharee, Lais S. Pereira, Safiatou Doumbo, Didier Doumtabe, Barbara J. Flynn, Arne Schön, Sachie Kanatani, Samantha O. Aylor, David Oyen, Rachel Vistein, Lawrence Wang, Marlon Dillon, Jeff Skinner, Mary Peterson, Shanping Li, Azza H. Idris, Alvaro Molina-Cruz, Ming Zhao, Lisa Renee Olano, Patricia J. Lee, Alison Roth, Photini Sinnis, Carolina Barillas-Mury, Kassoum Kayentao, Aissata Ongoiba, Joseph R. Francica, Boubacar Traore, Ian A. Wilson, Robert A. Seder, Peter D. Crompton

**Affiliations:** 1 Antibody Biology Unit, Laboratory of Immunogenetics, National Institute of Allergy and Infectious Diseases (NIAID), National Institutes of Health (NIH); Rockville, MD 20852, USA; 2 Malaria Infection Biology and Immunity Section, Laboratory of Immunogenetics, National Institute of Allergy and Infectious Diseases, National Institutes of Health; Rockville, MD 20852, USA; 3 Department of Integrative Structural and Computational Biology, Scripps Research Institute; La Jolla, CA 92037, USA; 4 Vaccine Research Center, National Institute of Allergy and Infectious Diseases, National Institutes of Health; Bethesda, MD 20892, USA; 5 Mali International Center of Excellence in Research, University of Sciences, Technique and Technology of Bamako; BP 1805, Point G, Bamako, Mali; 6 Department of Biology, Johns Hopkins University; Baltimore, MD 21218, USA; 7 Department of Molecular Microbiology & Immunology, Johns Hopkins Bloomberg School of Public Health; 615 N. Wolfe Street, Baltimore, MD 21205, USA; 8 Department of Drug Discovery, Experimental Therapeutics Branch, Walter Reed Army Institute of Research; Silver Spring, MD 20910, USA; 9 Biological Engineering Department, Massachusetts Institute of Technology; Cambridge, MA 02139, USA; 10 Laboratory of Malaria and Vector Research, National Institute of Allergy and Infectious Diseases, National Institutes of Health; Rockville, MD 20852, USA; 11 Protein Chemistry Section, Research Technologies Branch, National Institute of Allergy and Infectious Diseases, National Institutes of Health; Rockville, MD 20852, USA; 12 The Skaggs Institute for Chemical Biology, The Scripps Research Institute; La Jolla, CA, 92037, USA

## Abstract

Immunoglobulin (Ig)A antibodies play a critical role in protection against mucosal pathogens. However, the role of serum IgA in immunity to non-mucosal pathogens, such as *Plasmodium falciparum*, is poorly characterized, despite being the second most abundant isotype in blood after IgG. Here, we investigated the circulating IgA response in humans to *Plasmodium falciparum* sporozoites that are injected into the skin by mosquitoes and migrate to the liver via the bloodstream to initiate malaria infection. We found that circulating IgA was induced in three independent sporozoite-exposed cohorts: individuals living in an endemic region in Mali, malaria-naïve individuals immunized intravenously with three large doses of irradiated sporozoites, and malaria-naïve individuals exposed to a single controlled mosquito bite infection. Mechanistically, we found evidence in an animal model that IgA responses were induced by sporozoites at dermal inoculation sites. From malaria-resistant individuals, we isolated several IgA monoclonal antibodies that reduced liver parasite burden in mice. One antibody, MAD2-6, bound to a conserved epitope in the N-terminus of the *P*. *falciparum* circumsporozoite protein, the dominant protein on the sporozoite surface. Crystal structures of this antibody revealed a unique mode of binding whereby two Fabs simultaneously bound either side of the target peptide. This study reveals a role for circulating IgA in malaria and identifies the N-terminus of the circumsporozoite protein as a target of functional antibodies.

## Introduction

Malaria remains an enormous public health threat among the world’s most vulnerable populations. In 2019 alone there were an estimated 229 million cases of malaria resulting in 409,000 deaths (1). Most deaths occur among children and pregnant women in sub-Saharan Africa and are caused by the *Plasmodium falciparum* parasite, the deadliest of the five *Plasmodium* species that infect humans (1). Infection begins when *Anopheles* mosquitoes inject a small number of sporozoites into the skin. Sporozoites rapidly migrate to the liver via the bloodstream and infect hepatocytes where they differentiate into merozoites and replicate without causing symptoms. After approximately 7 days, merozoites rupture out of hepatocytes into the bloodstream and infect and replicate within erythrocytes in a 48-hour cycle that rapidly increases the number of blood-stage parasites and causes an acute, potentially life-threatening illness. In endemic areas, clinical immunity that protects from blood-stage disease can be acquired after years of repeated infections, although sterilizing immunity that prevents the progression to blood-stage infection appears to be rare (2, 3). The development of resistance to antimalarial medicines and insecticides is a perpetual challenge that jeopardizes efforts to control and eliminate malaria. The most clinically advanced malaria vaccine candidate, RTS,S, conferred up to 56% protection from clinical malaria after the first year and up to 36% protection at 3-4 years in those who received a booster (4–6), underscoring the need to develop additional tools and approaches by examining different facets of immunity against the malaria parasite.

Antibodies can play a critical role in mediating protection against all stages of malaria (7). The vast majority of studies on the antibody response to *P. falciparum* have focused on the immunoglobulin (Ig)G or IgM isotypes (7–11) based on the fact that most neutralization of *P*. *falciparum* occurs in the blood. In contrast, IgA antibodies are thought to play a much larger role in protection against mucosal pathogens (12). However, IgA is the second most abundant antibody isotype in blood after IgG, and the role of circulating IgA is poorly characterized (13). To investigate whether circulating IgA aids in defense against non-mucosal pathogens, we studied the IgA response to *P*. *falciparum* whose life cycle in humans is limited to non-mucosal organs. We examined the IgA response to *P*. *falciparum* sporozoites in individuals living in a malaria-endemic region of Mali, as well as malaria-naïve individuals who were immunized i.v. with radiation-attenuated sporozoites or exposed to infectious mosquito bites in a controlled setting. We isolated *P*. *falciparum* circumsporozoite protein (PfCSP)-specific IgA monoclonal antibodies (mAbs) from two Malian individuals and investigated the fine specificity and function of these antibodies. We found that exposure to sporozoites through different routes triggered a specific IgA response. We identified an IgA mAb, MAD2-6, that bound to a conserved epitope on the N-terminus of PfCSP, inhibited sporozoite invasion in vitro and reduced liver parasite burden in vivo. These findings reveal a role for IgA in the immune response to *P*. *falciparum* and identify the N-terminus of PfCSP as a target of functional antibodies.

## Results

### Infection and vaccination with *P*. *falciparum* sporozoites induce IgA antibodies that target PfCSP

**Fig. 1:**
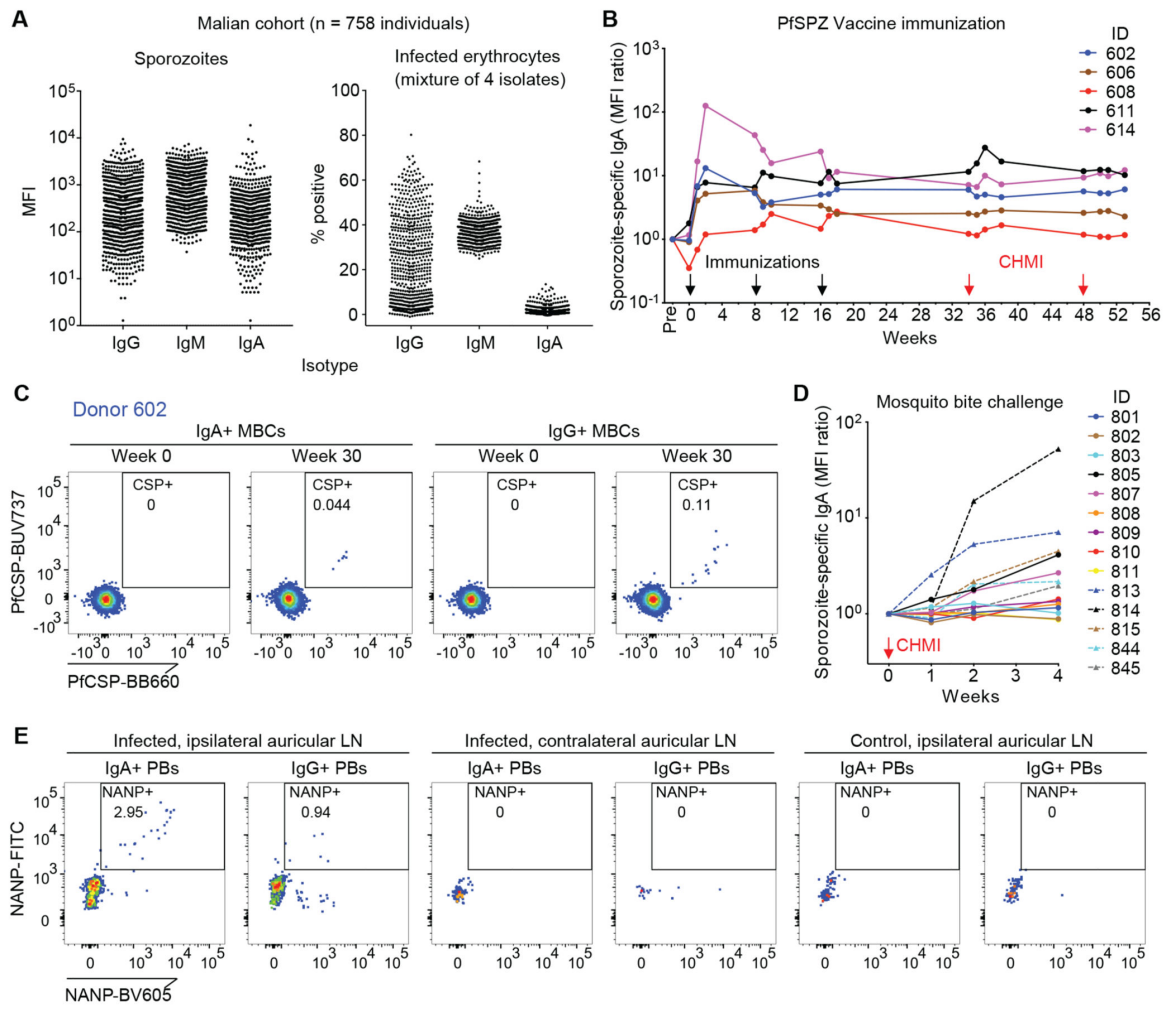
Infection and vaccination with *P*. *falciparum* sporozoites induces a long-lasting IgA response. (A) Binding of plasma IgG, IgM and IgA to *P*. *falciparum* sporozoites and infected erythrocytes among 758 Malian individuals (N = 2 experiments for sporozoites, N = 1 for infected erythrocytes). MFI, median fluorescence intensity. (B) Sporozoite-specific plasma IgA responses in U.S. volunteers after immunization with 3 x 900,000 cryopreserved, irradiated *P. falciparum s*porozoites (Sanaria PfSPZ Vaccine) (N = 2). MFIs were normalized to pre-immunization values. CHMI, controlled human malaria infection. ID, patient identification number. (C) PfCSP-specific IgA^+^ and IgG^+^ memory B cells (MBCs) in a sporozoite-immunized donor (Donor 602). Gated on live CD19^+^CD14^-^CD3^-^CD8^-^CD56^-^CD21^+^CD27^+^IgA^+^/IgG^+^ cells, with CD27^++^CD38^++^ plasmablasts excluded. (D) Sporozoite-specific plasma IgA responses in malaria-naïve U.S. volunteers after CHMI with 5 *P*. *falciparum*-infected mosquitos (N = 2). MFIs were normalized to pre-immunization values. (E) NANP-specific IgA plasmablasts from mice injected intradermally with transgenic *P*. *berghei* sporozoites expressing PfCSP (Pb-PfCSP) (n = 3 mice per group, N = 2 independent experiments). Cells isolated from indicated lymph nodes (LN) were analyzed 6 days after sporozoite injection. Gated on live CD138^+^IgA^+^/IgG^+^ cells. Control mice were injected with salivary gland extract from an equivalent number of mosquitoes used to obtain the sporozoites.

We first examined the IgA response to natural *P*. *falciparum* infection in a cohort study conducted in a region of Mali where the annual malaria season is clearly defined (fig. S1A). To detect long-lasting antibodies, we screened plasma collected from 758 individuals after the 5-month dry season (a period of negligible malaria transmission) for binding to *P*. *falciparum* NF54 sporozoites and a mixture of infected erythrocytes representing four parasite strains. In contrast to muted IgA responses to infected erythrocytes, many plasma samples showed IgA binding to sporozoites (Fig. 1A). We observed a significant correlation (r = 0.5426, P < 0.0001) between IgA binding to *P*. *falciparum* sporozoites and PfCSP (fig. S1B), consistent with the immunodominance of PfCSP on the sporozoite surface. We found a weaker correlation between IgA and IgG binding to sporozoites (r = 0.2627, P < 0.0001) (fig. S1B). Segmented regression analysis of the relationship between sporozoite-specific IgA and age revealed a breakpoint at 12.5 years (fig. S1B), and correlation analysis before and after this breakpoint showed that IgA binding to sporozoites increased slightly up to about 12 years of age (r = 0.2133, P < 0.0001) and then plateaued (r = -0.024, P = 0.670) (fig. S1B). To investigate if acute *P*. *falciparum* infection boosts IgA responses to sporozoites in previously infected individuals, we analyzed 10 Malian children aged 7 to 8 years with confirmed prior infections for their sporozoite-specific IgA, IgG and IgM response before, during and after a febrile malaria episode (fig. S1C). Although the effect was more pronounced for IgG and IgM, we found that sporozoite-specific IgA increased in 6 of 10 individuals in response to infection. To estimate the relative frequency of PfCSP-specific IgA^+^ and IgG^+^ memory B cells in malaria-experienced individuals, we isolated and polyclonally activated the respective B cell subsets from 50 Malian individuals and screened the supernatants for binding to PfCSP and Pf merozoite surface protein-1 (MSP-1) as a blood-stage antigen comparator. Of the 1.5 x 10^6^ IgA^+^ (598 wells) and 7.0 x 10^6^ IgG^+^ (2720 wells) memory B cells screened, we identified 37 positive PfCSP-specific IgA wells (6.2% of wells) and 96 positive PfCSP-specific IgG wells (3.5% of wells) (fig. S1D). In contrast, for PfMSP-1 we identified 29 positive IgA wells (4.8%) and 826 positive IgG wells (30.4%). Collectively, these data suggest that natural *P*. *falciparum* infection can trigger the generation of anti-sporozoite IgA antibodies and PfCSP-specific memory B cells.

We also measured sporozoite-specific IgG, IgM and IgA in plasma samples of 43 malaria-naïve U.S. adults (fig. S2A). Several individuals had high amounts of sporozoite-specific IgG and overall, these individuals had amounts of IgA and IgM binding to sporozoites that were comparable to the Malian responses, presumably due to pre-existing B cells primed by non-*Plasmodium* antigens whose antibodies cross-react with sporozoite surface antigens (14). To determine if exposure to sporozoites triggers a specific IgA response in malaria-naïve individuals, we screened longitudinal plasma samples from a clinical trial (VRC 314) in which malaria-naïve U.S. individuals were immunized intravenously (i.v.) with three doses of irradiated, aseptic, cryopreserved *P*. *falciparum* NF54 sporozoites (Sanaria PfSPZ Vaccine) prior to controlled human malaria infection (CHMI) with *P*. *falciparum* 3D7-infected mosquitoes. The PfSPZ Vaccine induced an IgA response against sporozoites and PfCSP (Fig. 1B, fig. S2B), consistent with a study reporting PfCSP-specific IgA in serum after immunization with sporozoites under chemoprophylaxis (14). The sporozoite-specific IgA targeted the immunodominant NANP repeat region and remained stably elevated in 4 of 5 individuals over a year that included a 4-month period without sporozoite exposure (fig. S2B), suggesting that long-lived IgA^+^ plasma cells were generated in these individuals. PfCSP-specific IgA^+^ memory B cells were also detected three months after the final immunization (Fig. 1C, fig. S2C and D).

We also examined malaria-naïve individuals who were exposed to the bites of 5 *P*. *falciparum* 3D7-infected mosquitos through CHMI without prior immunization. Exposure to a single infection dose induced sporozoite-specific plasma IgA, with 6 of 14 individuals evaluated reaching a greater than 2-fold increase in antibody binding (Fig. 1D), confirming that the natural route of sporozoite transmission induces IgA antibodies. As expected, we also observed an anti-sporozoite IgG response in the PfSPZ Vaccine-immunized and infected mosquito-exposed individuals. In the immunized individuals, the IgG response peaked later and plateaued at a higher baseline than IgA (fig. S2E), whereas the group exposed only to *P*. *falciparum*-infected mosquitos displayed a similar IgG response to IgA in terms of the specific individuals that responded, as well as the kinetics and magnitude of the response (fig. S2F).

To determine the initial site of IgA class switching after sporozoite exposure, we used a mouse model that allowed us to control the site of sporozoite delivery. We injected the left ear of BALB/c mice intradermally with transgenic *P*. *berghei* sporozoites that express PfCSP in place of *P*. *berghei* CSP (Pb-PfCSP). Six days later, NANP-specific IgA^+^ plasmablasts were detected in the draining ipsilateral auricular lymph node (LN) and spleen, but not in the contralateral auricular LN, portal/celiac LNs, mesenteric LNs or Peyer’s patches (Fig. 1E, fig. S3), the latter two being intestinal sites traditionally associated with IgA^+^ B cell class switching. Control mice injected with salivary gland extract from uninfected mosquitoes did not generate NANP-specific responses (Fig. 1E). These findings are consistent with reports of sporozoite entry into lymph nodes draining skin inoculation sites (15) and suggest that sporozoites induce IgA responses at these locations.

### Isolation and characterization of IgA monoclonal antibodies targeting PfCSP

**Fig. 2:**
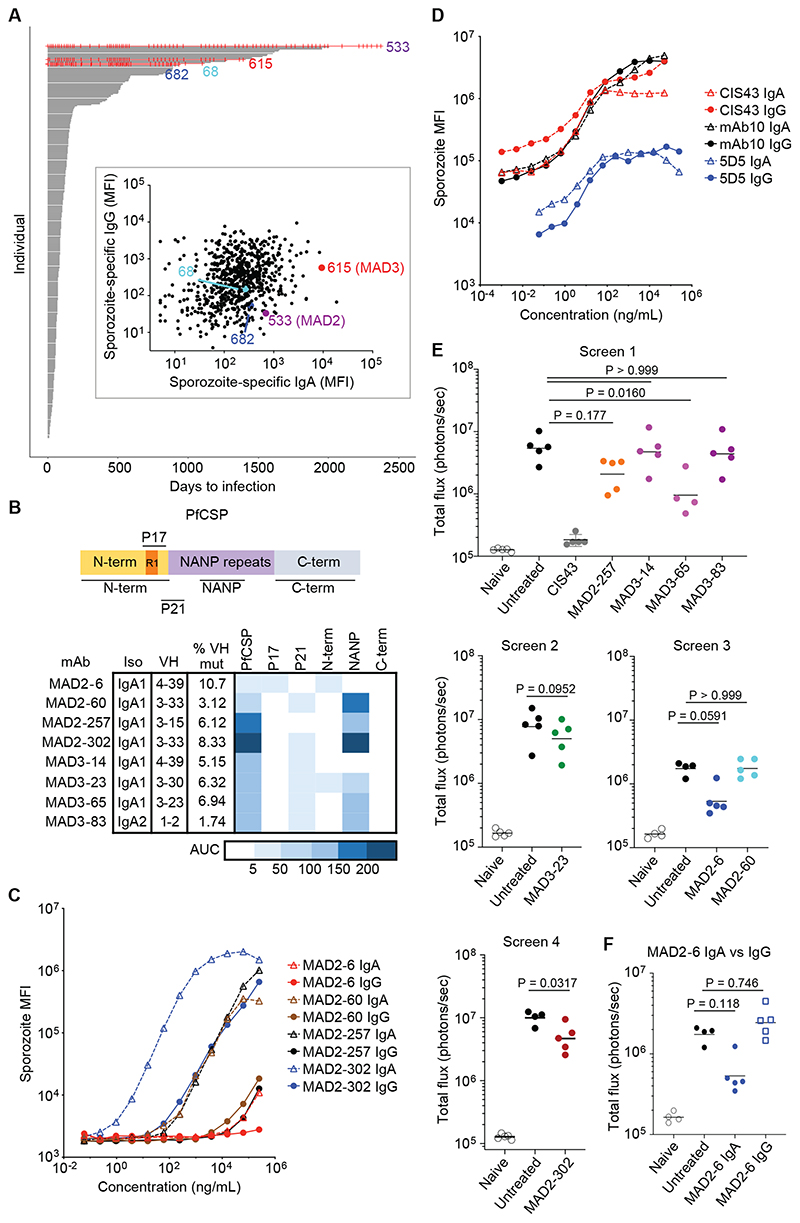
Naturally acquired IgA monoclonal antibodies reduce sporozoite infection in vivo. (A) Time to PCR-positive *P*. *falciparum* blood-stage infection since study enrollment. Each individual in the cohort (n = 758 individuals) is represented by a horizontal line that ends when they test positive under bi-weekly or monthly active surveillance by fingerprick sampling, irrespective of symptoms. Individuals who were never PCR-positive are shown in red (three red lines stopped early when those individuals left the study). Each red vertical mark indicates when a PCR test was performed. Inset shows sporozoite-specific plasma IgG versus IgA for the cohort, with the four PCR-negative individuals highlighted. MFI, median fluorescence intensity. (B) Schema of PfCSP and binding of IgA monoclonal antibodies isolated from donors MAD2 and MAD3 to peptides from different regions of PfCSP. The heat map shows area under the curve (AUC) from a titration of each antibody with each peptide (N = 2). (C) Binding of IgA or IgG monoclonal antibodies from MAD2 (originally isolated as IgA) to *P*. *falciparum* sporozoites is shown (N = 2). Antibody binding was detected using the same secondary anti-light chain antibody to allow comparison between the isotypes. (D) Sporozoite binding of IgA versus IgG monoclonal antibodies, previously isolated as IgG (N = 2). (E) In vivo screen of IgA monoclonal antibodies (n = 5 mice per group, except for MAD3-65, where n = 4). Mice were injected i.v. with 300 μg of antibody and challenged i.v. with 2,000 Pb-PfCSP. Parasite liver burden was measured by IVIS 2 days later. Horizontal bars show geometric mean. The native deglycosylated form of MAD2-6 IgA was used in this assay. Differences in parasite liver burden between each antibody and the untreated control were determined using a Kruskal-Wallis test with Dunn’s multiple comparison correction for panels with multiple antibodies and a two-tailed Mann-Whitney test for the panel with MAD2-302 and MAD3-23. CIS43, a highly potent antibody that targets the junction and NANP repeats (17), was used as a positive control. (F) Comparison of MAD2-6 IgA and IgG in an in vivo assay (n = 5 mice per group). The IgG was tested in the same experiment as screen 3 and shown here separately for clarity, with the other treatment groups shown again. Horizontal bars show geometric mean. The native deglycosylated form of MAD2-6 IgA was used in this assay. Differences in parasite liver burden between each antibody and the untreated control were determined using a Kruskal-Wallis test with Dunn’s multiple comparison correction.

To study the human IgA response at higher resolution, we established a protocol to isolate IgA anti-CSP mAbs from individuals in the Mali cohort. We downselected donors based on two criteria. First, we identified donors who were *P*. *falciparum* blood-stage negative by polymerase chain reaction (PCR) every time that they were tested as a possible indication of resistance to sporozoite infection. Of the 758 individuals followed by active surveillance for infection, only four met this criterion (Fig. 2A). Second, we identified donors with serologic evidence of sporozoite exposure, and more specifically, examined *P*. *falciparum* sporozoite-specific plasma IgA of the four PCR-negative individuals relative to the rest of the cohort. Based on these criteria, we chose two donors: MAD2, who tested negative by PCR 81 consecutive times over seven years; and MAD3, who tested negative by PCR for 4 years and had the second-highest anti-sporozoite IgA in the cohort (Fig. 2A). Approximately 35,000 IgA^+^ B cells from MAD2 and 56,000 IgA^+^ B cells from MAD3 were screened for antibodies that bound freshly dissected *P*. *falciparum* NF54 sporozoites and recombinant PfCSP. We sequenced the antibody heavy and light chains from positive B cells and identified eight recombinant IgA mAbs with confirmed binding to PfCSP and sporozoites from the two donors (Fig. 2B, table S1). Somatic (nucleotide) mutations in the VH genes of these antibodies ranged from 1.7 to 10.7%, consistent with antibody affinity maturation during germinal center reactions. Seven of eight antibodies were of the IgA1 isotype, which was unsurprising given the dominance of this isotype in circulation. We expressed the antibodies in their original IgA1 or IgA2 isotype without the J-chain to allow production of monomers, which is the main IgA form in blood.

We mapped the binding sites of these antibodies using peptides covering different regions of PfCSP (table S2), as previously described (16, 17). Six antibodies bound to the immunodominant NANP repeat region (Fig. 2B), and three of these were encoded by the *VH3-30/VH3-33* lineage commonly seen in PfCSP-specific IgG antibodies (14, 16, 18). Interestingly, antibody MAD2-6 bound to a peptide P17 that covers region I (KLKQP), which is a conserved functional site in the N-terminus of PfCSP associated with cleavage prior to hepatocyte invasion (19). We confirmed binding of this antibody to live sporozoites by flow cytometry (fig. S4A and B). To determine whether the IgA isotype is important for binding, we replaced the IgA1/IgA2 backbone with IgG1 and compared their binding to *P*. *falciparum* sporozoites, recombinant PfCSP, and the relevant peptides using anti-light chain secondary antibodies. Although this assay was conducted on monomeric forms of both isotypes, most of the mAbs bound better to *P*. *falciparum* sporozoites and PfCSP as IgA compared to IgG (Fig. 2C, fig. S4C and D). In contrast, when we converted previously characterized anti-PfCSP IgG mAbs (17, 20) to IgA, binding was largely unchanged (Fig. 2D). Taken together, these results suggest that IgA^+^ B cells undergo class switching and affinity maturation in germinal center reactions, and that the IgA Fc region influences antigen binding of native IgA antibodies.

### MAD2-6 IgA targets the N-terminus of PfCSP and is functional in vivo

Next, we tested whether the IgA mAbs could protect in vivo by injecting them into C57BL/6 mice, challenging the mice i.v. with Pb-PfCSP sporozoites, and measuring parasite liver burden two days later. Initial screens with 4 to 5 mice per group revealed that two IgA antibodies, MAD2-6 and MAD3-65, resulted in the greatest reduction in parasite burden (Fig. 2E). MAD3-65 binds to the NANP repeat region, which is a known target of potent anti-PfCSP antibodies. However, the protective effect of MAD2-6, which targets the PfCSP N-terminus (Fig. 2B), was surprising given its relatively poor binding to sporozoites (Fig. 2C). Thus, we focused subsequent in vivo experiments on MAD2-6. In vivo experiments with IgA proved technically challenging due to rapid clearance of human IgA in mice (21, 22) (fig. S5A). We found that deglycosylation of the tailpiece N-glycan by treatment with PNGaseF increased antibody stability in vivo to a degree comparable to a human IgG mAb for the first 10 hours (fig. S5A). Consequently, only MAD2-6 IgA deglycosylated with PNGaseF, or through spontaneous deglycosylation after storage at 4°C, showed protective efficacy in mice (fig. S5B to D). Although the kinetics of MAD2-6 IgG were comparable to deglycosylated MAD2-6 IgA, IgG treatment showed no protective efficacy in vivo (Fig. 2F).

**Fig. 3:**
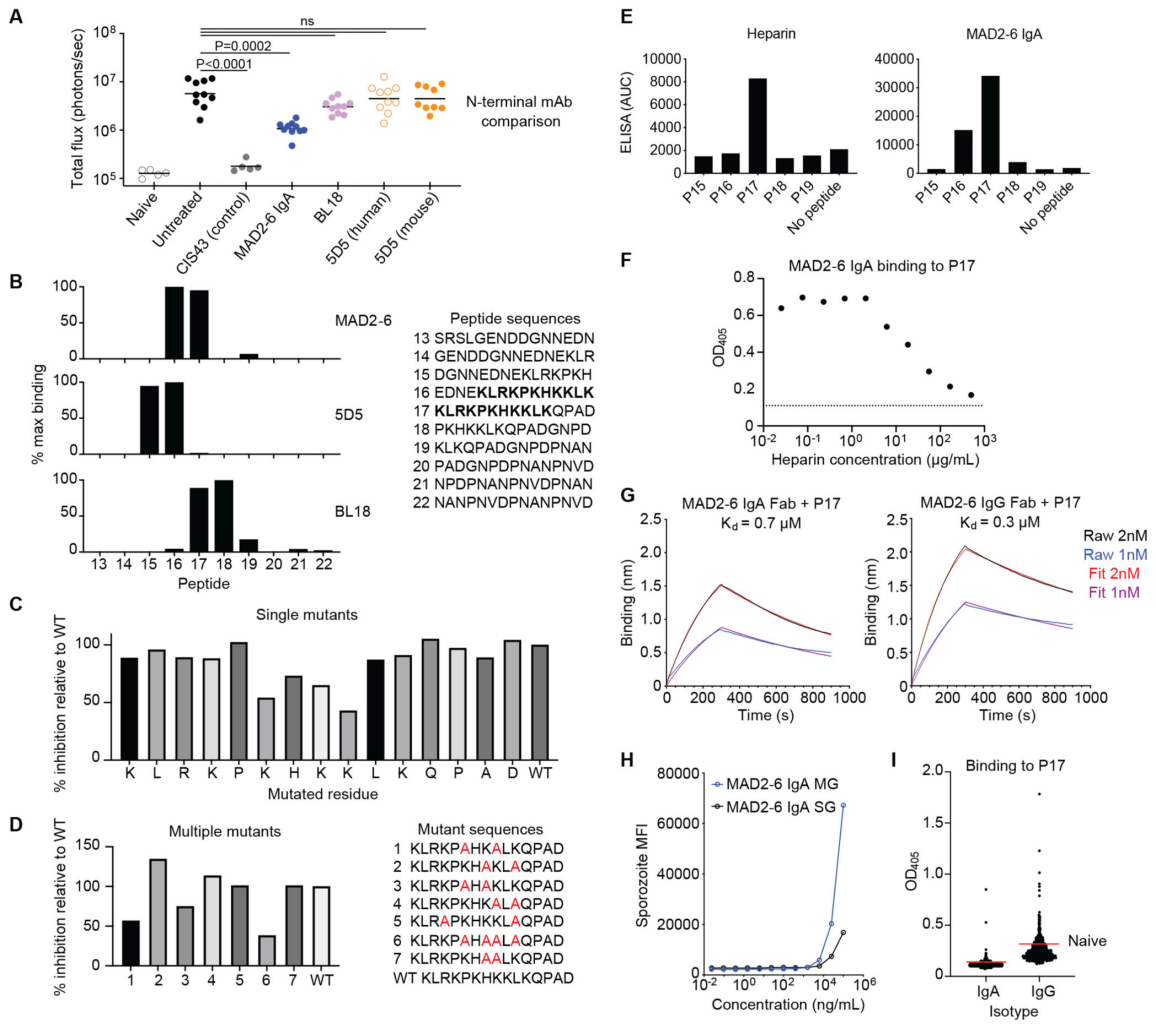
MAD2-6 binds to a unique conserved region in the N-terminus of CSP (A) Comparison of efficacy between mAb 5D5 (mouse and human), mAb BL18 and deglycosylated MAD2-6 IgA in an in vivo liver burden reduction assay (n = 10 mice per group). Differences in liver burden between each antibody and the untreated (no antibody) control was determined using a Kruskal-Wallis test with Dunn’s multiple comparison correction. Horizontal bars show geometric mean. mAb CIS43 is a positive control antibody. (B) Binding of MAD2-6 IgA, 5D5 and BL18 to peptides from the PfCSP N-terminus (N = 2). Binding was measured by fluorescence intensity in a multiplex plate-based assay and normalized to the strongest signal for each antibody. The overlapping sequence in the two peptides bound by MAD2-6, peptides 16 and 17, is shown in bold. (C) Competition ELISA with P17 mutants carrying a single alanine substitution at each position (N = 2). Each bar shows the relative ability of a mutant peptide to compete with the wild-type (WT) peptide in binding to MAD2-6 IgA. (D) Competition ELISA with P17 mutants carrying multiple alanine substitutions at different lysine residues (N = 2). Each bar shows the relative ability of a mutant peptide to compete with the WT peptide in binding to MAD2-6 IgA. (E) Binding of heparin and MAD2-6 IgA to PfCSP N-terminus peptides, as measured by ELISA (N = 2). AUC, area under the curve. (F) Binding of MAD2-6 IgA to P17 with competition from heparin, as measured by ELISA (N = 2). The dotted line shows the baseline signal with no peptide added. OD_405_, optical density at 405nm. (G) Biolayer interferometry (Octet) showing MAD2-6 IgA Fab or MAD2-6 IgG Fab binding to P17 (N = 2). K_d_, dissociation constant. The Raw curves connect all data points, and the Fit curves are based on a model of 1:1 binding calculated using the Octet Data Analysis software. (H) Binding of MAD2-6 IgA to midgut (MG) and salivary gland (SG) *P*. *falciparum* sporozoites (N = 2). MFI, median fluorescence intensity. (I) Binding of plasma IgG, IgM and IgA among 758 Malian individuals to peptide P17 (N = 1). The red lines show the mean binding based on OD_405_ of eight malaria-naïve U.S. adults.

Given the potential importance of this N-terminal site as a neutralizing epitope, and the fact that it is completely conserved in >2000 *P*. *falciparum* isolates (analyzed with Panoptes Pf3k), we extended the in vivo analysis to compare MAD2-6 with other N-terminal antibodies in a definitive experiment with 9 to 10 mice per group. It has proven difficult to isolate mAbs against the N-terminus, with only a small number of mouse antibodies isolated to date. To our knowledge, mouse antibody 5D5 is the only other mAb targeting the N-terminus that has been tested in vivo, with variable efficacy reported in different studies (20, 23). Furthermore, we previously isolated a rhesus IgG mAb, BL18, that binds to region I. We compared these antibodies for in vivo efficacy using the Pb-PfCSP sporozoite challenge model and found that, although MAD2-6 IgA was not as potent as the positive control antibody CIS43 (17), it was the only N-terminal-specific antibody that significantly (P = 0.0002) reduced parasite burden (Fig. 3A).

### MAD2-6 IgA binds to a conserved site within the PfCSP N-terminus

To investigate the mechanism underlying their differential potency, we mapped the binding sites of MAD2-6, 5D5 and BL18 using overlapping peptides that cover the sequence proximal to region I. MAD2-6 binds to a lysine-rich region between the binding sites of 5D5 and BL18 (Fig. 3B). Alanine scan mutagenesis revealed that these basic amino acids, specifically the **K**H**KK**L**K** motif overlapping with region I, are important for MAD2-6 binding (Fig. 3C, fig. S6A). Nevertheless, some binding was retained with double and even quadruple lysine mutations, suggesting that the remaining basic amino acids contribute to binding and compensate for loss of the core motif (Fig. 3D). This lysine-rich site has been linked to PfCSP binding to heparan sulfates on hepatocytes during sporozoite attachment (24, 25). We confirmed by enzyme-linked immunosorbent assay (ELISA) that heparin mirrors the binding pattern of MAD2-6 and binds most strongly to P17 (Fig. 3E), and that MAD2-6 and heparin compete for binding to this peptide (Fig. 3F and fig. S6B). Affinity measurements by isothermal titration calorimetry and biolayer interferometry showed that MAD2-6 IgA and IgG bind to PfCSP and P17 with comparable micromolar affinity (Fig. 3G, fig. S6C and D). Thus, the protective activity of MAD2-6 IgA in vivo is perhaps unexpected given its relatively low affinity for PfCSP and weak binding to sporozoites in vitro. One potential explanation is that MAD2-6 binds more strongly to a transitional conformation of PfCSP displayed during hepatocyte invasion. To test this hypothesis, we measured binding of MAD2-6 to *P*. *falciparum* mosquito midgut sporozoites, which display PfCSP in an open conformation that is thought to be similar to its conformation during hepatocyte invasion (26). Both MAD2-6 IgA and MAD2-6 IgG bound better to midgut than salivary gland *P*. *falciparum* sporozoites at the highest concentration tested (Fig. 3H, fig. S7A). In contrast, a control anti-NANP repeat antibody bound two-fold better to salivary gland sporozoites at the highest concentration tested (fig. S7B), suggesting that the improved binding of MAD2-6 to midgut sporozoites was not due to higher expression of surface PfCSP. To test other potential ways that MAD2-6 may interact with sporozoites, we assessed its function in several in vitro assays. MAD2-6 IgA did not trigger the CSP reaction (fig. S7C and D) or impact *P*. *falciparum* sporozoite gliding motility (fig. S7E to G), unlike the control anti-NANP repeat antibody 2A10. However, MAD2-6 IgA inhibited sporozoite invasion of hepatocytes and subsequent development of liver stage schizonts (day 5 post-infection) by about 90% at a concentration of 200 μg/mL (fig. S7H and I), consistent with its activity in vivo.

We investigated whether the N-terminal epitope targeted by MAD2-6 was commonly recognized by plasma from the Mali cohort. We observed a variable IgA and IgG response to the P17 peptide, with the majority of individuals (87% and 81% for IgA and IgG, respectively) not showing stronger antibody binding than malaria-naïve U.S. individuals (Fig. 3I). In age-adjusted analysis we found no correlation between plasma IgA binding to peptide 17 and the risk of febrile malaria; however, for every 24% increase in IgG binding to peptide 17, there was a 50% reduction in the number of febrile malaria episodes (p = 0.01 after Bonferroni correction), suggesting that the MAD2-6 N-terminal epitope may be a relevant target of naturally acquired immunity to malaria.

### Structural basis for recognition of the N-terminal epitope by MAD2-6 IgA Fab

**Fig. 4:**
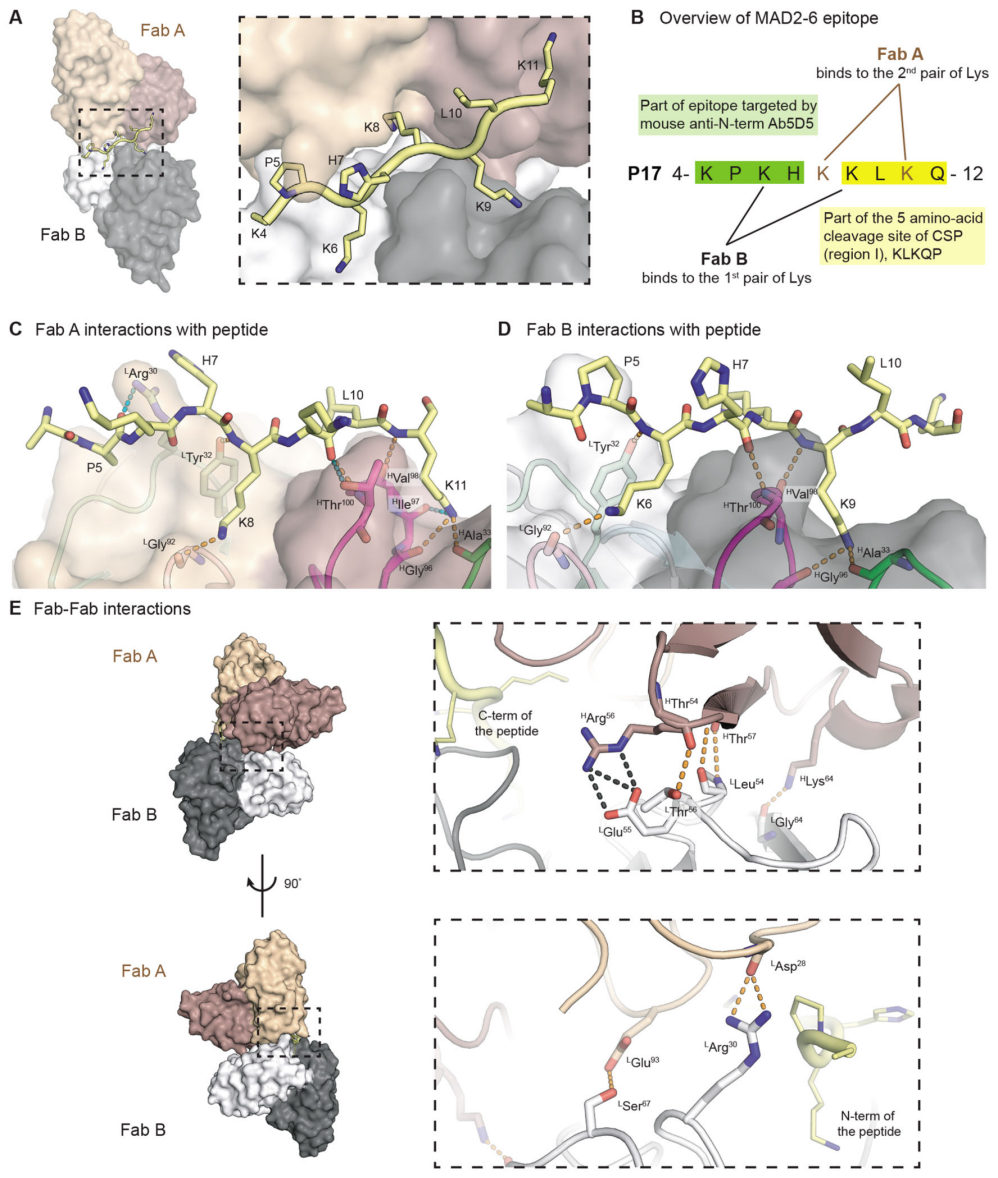
Structural basis for recognition of the N-terminal epitope containing region I by MAD2-6 IgA Fab. (A) The variable domain of Fab A (heavy chain: brown; light chain: light brown) and Fab B (heavy chain: black; light chain: grey) are shown as surfaces. P17 peptide is displayed in ribbon representation with side chains as sticks. The dashed box indicates the enlarged area. Peptide residue numbers are shown. (B) Overview of MAD2-6 epitope and Fab A and Fab B binding. (C) and **(D)** Interactions of MAD2-6 IgA Fabs A (C) and B (D) with part of the P17 peptide. Fabs are displayed in ribbon representation overlayed with transparent surfaces. CDRs are colored light green, light cyan, pink, magenta, and green for CDR L1, L2, L3, H3, and H1, respectively. Hydrogen bonds (dashed lines) that are shared in both Fabs A and B are shown in orange, whereas those unique to each Fab are shown in teal. The peptide is represented as yellow sticks. Fab residues number are shown with superscript H and L for heavy and light chain, respectively. (E) Homotypic interactions of two MAD2-6 IgA Fabs. The Fab variable domains are shown as surfaces and ribbon representation in the enlarged box with the same coloring scheme for heavy and light chains as in (A). Black and orange dashes represent salt bridges and hydrogen bonds, respectively.

To ascertain how MAD2-6 recognizes the lysine-rich site at the PfCSP N-terminus, crystal structures of MAD2-6 IgA and IgG Fabs were determined in complex with short peptides that contain portions of P17 (KPKHKKLKQ for IgA Fab and EKLRKPKHKKLKQ peptide for IgG Fab) at 2.5 and 2.1 Å resolution, respectively (Fig. 4, fig. S8, and table S3). For simplicity, the peptides in both Fabs are numbered according to the full sequence of P17. Interestingly, two copies of the Fab in both structures simultaneously recognize two different pairs of lysines, which are found in two overlapping KxxK motifs; each Fab binds on either side of the peptide (Fig. 4A and B, fig. S8A and B), consistent with the redundancy observed in the peptide mutagenesis competition ELISA. The epitope targeted by MAD2-6 contains part of the epitope targeted by 5D5 as well as up to four residues from the region I KLKQP sequence (Fig. 4B) (25). The Fab paratope is composed of complementarity-determining region (CDR) heavy (H)1, H3, light (L)1, and L3, which form two binding pockets that accommodate Lys8 and Lys11 in one Fab and Lys6 and Lys9 in the other Fab (Fig. 4C and D). The interactions between each IgA Fab and their epitopes are almost identical: hydrogen bonds are made between the side chain of the first Lys in the KxxK motif and the CDR L3 backbone, and between the side chain of the second Lys and the CDR H1 and H3 backbone (Fig. 4 C and D). Similar pairs of peptide backbone hydrogen bonds are made to the backbone of ^H^Gly^96^, ^H^Val^98^, and ^H^Thr^100^ in CDR H3, as well as to the side chain of ^H^Thr^100^ in Fab A (Fig. 4C and D). Unique hydrogen bonds are also formed between ^L^Arg^30^ of Fab A and the Pro5 mainchain (Fig. 4C). Additional electron density for the bound peptide (slightly longer, see Methods) was observed in the MAD2-6 IgG Fab structure, which includes Gln12 from region I, allowing detection of additional hydrogen bonds between Fab B and the peptide termini. The difference in the number of hydrogen bonds to the overlapping epitope (from K4-K11) between IgG and IgA Fabs is likely due to the higher resolution of the IgG Fab structure. Nonetheless, the region that interacts with both MAD2-6 IgA and IgG Fabs, from Lys4-Lys11, adopts a virtually identical conformation and Fab interactions (fig. S8C to E). Additionally, since the two copies of either MAD2-6 IgA or IgG Fabs come into close contact while simultaneously binding the peptide, they display homotypic Fab-Fab interactions, which were first observed in anti-NANP antibodies (27–29). These interactions are mainly between the heavy chain of one Fab and light chain of the other Fab and include extensive hydrogen bonds and salt bridges between CDRs H2 and L2 (Fig. 4E, fig. S8C, fig. S9). Most key interacting light-chain residues are present in the germline gene, whereas some of the key heavy-chain residues are somatically mutated (fig. S9). Thus, MAD2-6 exhibits an inherent propensity for homotypic Fab-Fab interactions that are further strengthened by heavy-chain somatic hypermutation, potentially explaining its low affinity in vitro and increased avidity in vivo. However, this avidity effect can be even more prominent for anti-NANP antibodies that simultaneously bind multiple epitopes formed by tandem NANP repeats (27, 29).

Although both MAD2-6 IgA and IgG Fabs exhibit identical epitope conformations, peptide interactions and homotypic contacts, some differences are observed in their constant domains. The elbow region sequence of IgA Fab, which connects V_H_ to the C_H_1 domain, differs from that of the IgG Fab and allows for formation of a hydrophobic core consisting of ^H^Leu^11^, ^H^Pro^116^, and ^H^Tyr^203^ (fig. S8F), as previously described (30). This hydrophobic core offers potentially increased rigidity to the elbow region and is absent from Fabs of other antibody isotypes (30). Furthermore, the IgA Fab possesses two unique disulfide bonds in the constant domains. The extra, intra-domain disulfide between ^H^Cys^214^ and ^H^Cys^190^ in C_H_1 may further stabilize this domain in the IgA, whereas another between the C-terminus of the C_L_ (^L^Cys^214^) to the middle of C_H_1 (^H^Cys^127^) could help stabilize the constant region (fig. S8G). A corresponding interchain disulfide between ^L^Cys^214^ and ^H^Cys^215^ in IgG is sometimes observed in Fab crystal structures, such as anti-NANP Ab 366 (29) (fig. S8G). Thus, differences in the elbow region and constant domains may translate to greater rigidity in IgA Fabs, and potentially contribute to stronger binding of MAD2-6 IgA to sporozoites, along with other factors, including higher flexibility in the IgA1 hinge region and Fc differences (31).

## Discussion

This study opens additional questions on the role of circulating IgA antibodies in host defense against non-mucosal pathogens. Whereas the IgA contribution to mucosal immunity is well-documented, the origin and function of serum IgA remain unclear. Since the energy expenditure required to maintain serum IgA rivals that of IgG (13), it is likely that circulating IgA confers a selective advantage. It is possible that serum IgA protects against bacteria that breach mucosal barriers (32). Consistent with this hypothesis, a proportion of human serum IgA targets bacteria that typically colonize mucosal surfaces (33–35). Serum IgA can be generated by long-lived IgA^+^ plasma cells of mucosal origin that migrate to bone marrow (36–38), or possibly as a result of leakage of bacterial antigens from the gut into systemic circulation, activating B cells (39). Here, we show a more direct function of circulating IgA where it contributes to protection against a non-mucosal bloodborne pathogen, which is historically attributed to IgG and IgM. These findings are consistent with a recent systems serology study reporting that IgA responses targeting the PfCSP NANP repeat region and C-terminus were associated with protection in trials of the malaria vaccine candidate RTS,S/AS01 (40). Our findings suggest that B cells can class switch to IgA at the site of antigen exposure in non-mucosal tissue and undergo affinity maturation through germinal center reactions, resulting in long-lived plasma cells that produce circulating IgA antibodies against *P*. *falciparum* sporozoites. It will be interesting to determine the full range of function of serum IgA by investigating analogous responses to other non-mucosal pathogens.

The discovery of MAD2-6 revealed an epitope on the PfCSP N-terminus that can be targeted by antibodies. All potent anti-PfCSP mAbs discovered to date bind to the dominant NANP repeat region, with a few antibodies binding to junctional epitopes that includes variations of the core NPNA motif (14, 16–18, 41). In contrast, very few antibodies targeting the N-terminal region have been isolated (20, 26), with a recent study (23) and our own data suggesting that the best-characterized N-terminal mouse antibody, 5D5, is not protective in vivo. However, binding of serum antibodies to a PfCSP N-terminal peptide correlated with protection in children living in a malaria-endemic area, suggesting that regions of the N-terminus can be targeted by protective antibodies (42). Our data showing a correlation between polyclonal P17-specific IgG in plasma and protection from clinical malaria support this conclusion. The MAD2-6 epitope has been implicated in binding to heparan sulfates on the hepatocyte surface (24, 25, 43) and in cleavage of the N-terminus to aid sporozoite invasion (19, 44, 45). The KxxK motif bound by MAD2-6 is strikingly similar to heparin/heparan sulfate consensus binding motifs (25) and we confirmed that MAD2-6 and heparin compete for binding to the P17 peptide.

This study has limitations. First, although rare individuals in the Mali cohort appear to be more resistant to *P*. *falciparum* infection, it is unknown whether the CSP-specific IgA monoclonal antibodies isolated from these individuals contribute to their apparent protection. Second, we observed an association between protection from febrile malaria and polyclonal IgG binding to the P17 peptide, though this was not observed for polyclonal IgA. Although this finding highlights the potential importance of P17 as a target of protective antibodies in general, further studies are needed to determine whether vaccine-induced or naturally acquired polyclonal IgA responses to sporozoite antigens contribute to protection. Third, we found that several malaria-naïve individuals show high IgG binding to sporozoites, and overall, these individuals have amounts of IgA and IgM binding to sporozoites that were comparable to those of malaria-exposed Malians. We speculate that some malaria-naïve individuals have memory B cells and plasma cells primed by non-*P*. *falciparum* antigens whose antibodies cross-react with sporozoite antigens. This hypothesis is consistent with a study reporting high mutation frequencies in PfCSP-specific B cell receptors in malaria-naïve individuals after their first exposure to sporozoites (14), as well as several reports that PfCSP-specific antibodies are often derived from the common VH gene family VH3-30/3-33 (14, 16, 18), an observation we also made in the present study. It will be of interest in future studies to compare the biology of sporozoite-specific memory B cells of various isotypes in malaria-naïve and experienced individuals in the context of infection and vaccination. Fourth, the precise inhibitory mechanisms of MAD2-6 IgA remain to be determined. It is possible that MAD2-6 IgA and other sporozoite-specific IgA antibodies exert some protective effect through Fc-mediated mechanisms. Because mice do not have the homolog of the human IgA Fc receptor (FcαRI/CD89), it is unlikely that the Fc region of MAD2-6 IgA offers any advantage in the mouse model used in this study. Future studies with mice transgenic for human FcαRI/CD89 could shed light on this question. Additionally, although we provide evidence that MAD2-6 IgA competes with heparin for binding to the P17 peptide, it will also be of interest to determine whether MAD2-6 IgA inhibits proteolytic processing of the N-terminal region of PfCSP (19). Finally, our findings in the mouse model suggest that sporozoites induce IgA responses in skin-draining lymph nodes, consistent with reports of sporozoite entry into these lymph nodes (15); however, further research is required to definitively determine the site at which IgA responses are induced by sporozoites.

In conclusion, we found that exposure to *P*. *falciparum* sporozoites through infection or immunization triggers a functional IgA response. Analysis of PfCSP-specific IgA mAbs from malaria-exposed individuals revealed that the antibodies targeted distinct regions of this protein. The antibody MAD2-6 IgA, which bound to a conserved site on the N-terminus of PfCSP, inhibited sporozoite invasion of hepatocytes in vitro and reduced liver parasite burden in vivo. Collectively, these data reveal a role for IgA in the immune response to malaria and support further exploration of the PfCSP N-terminus as a target of protective antibodies.

## Materials And Methods

### Study design

The cohort study was conducted in the rural village of Kalifabougou, Mali where *P*. *falciparum* transmission occurs primarily from June to December each year. The cohort has been described in detail elsewhere. Briefly, since 2011, 1187 subjects aged 1 month to 43 years have been enrolled in this ongoing study (46), and 50.8% of subjects are female. The names of potential subjects are selected at random (computer-generated randomization) from an age- and gender-stratified census (previously collected) of the entire village population of approximately 5000 inhabitants. The study team contacts selected individuals in person by visiting their respective homes and inviting them to participate in the study. Exclusion criteria at the time of screening include anemia (hemoglobin <7 g/dL); current use of antimalarials, corticosteroids, or other immuno-suppressants; underlying heart disease, bleeding disorder, or other conditions that, in the judgment of the clinical investigators, could increase the risk to the study subjects; fever >37.5°C or evidence of an acute infection; or currently pregnant or planning to become pregnant during the study period. There is no exclusion based on race, ethnicity, sex or gender. Clinical malaria episodes were detected prospectively by self-referral and weekly active surveillance visits. All individuals with signs and symptoms of malaria and any *Plasmodium* parasitemia detected by light microscopy were treated according to the National Malaria Control Program guidelines in Mali. In addition, every two to four weeks during scheduled visits, blood was collected by finger prick from all subjects to retrospectively detect asymptomatic *P*. *falciparum* infections by microscopic examination of blood smears and polymerase chain reaction (PCR) analysis of dried blood spots (DBS) on filter paper (Protein Saver 903, Whatman). Venous blood samples were collected before and after each malaria season, at the time of diagnosis of the first clinical malaria episode of each malaria season, and 7-10 days later. Venous blood was drawn into sodium citrate-containing cell preparation tubes (BD Biosciences) and peripheral blood mononuclear cells (PBMCs) and plasma were isolated within 2 hours according to the manufacturer’s instructions. Plasma and PBMCs were stored at −80°C and in vapor-phase liquid nitrogen, respectively. For this study, we focused on 758 subjects who had plasma and PBMCs available for analysis. Monoclonal antibodies were isolated from two individuals in the cohort based on clinical and serological data as described in the text, and all IgA monoclonal antibodies that bound to both *P*. *falciparum* sporozoites and PfCSP from these two individuals were characterized in this study. For mAb analysis, no randomization or blinding was performed.

### Ethics approval

The Kalifabougou cohort study was approved by The Ethics Committee of the Faculty of Medicine, Pharmacy and Dentistry at the University of Sciences, Technique and Technology of Bamako, and the Institutional Review Board of the National Institute of Allergy and Infectious Diseases, National Institutes of Health (NIH IRB protocol number: 11IN126; https://clinicaltrials.gov/; trial number NCT01322581). Written informed consent was obtained from participants or parents or guardians of participating children before inclusion in the study.

### Isolation of IgA monoclonal antibodies

Cryopreserved PBMCs from the Malian cohort were thawed and stained for 20 min at 4˚C in phosphate buffered saline (PBS) + 1% fetal bovine serum (FBS) with the following panel: 1:400 LIVE/DEAD Fixable Aqua, 1:50 CD14-Brilliant Violet (BV)510 (BioLegend 301842), 1:50 CD3-BV510 (BioLegend 317332), 1:50 CD56-BV510 (BioLegend 318340), 1:25 CD19-Electron Coupled Dye (ECD) (Beckman Coulter IM2708U), 3 μg/mL IgA-Alexa Fluor 647 (Jackson Immunoresearch 109-606-011), 1:125 IgD-phycoerythrin-Cyanine 7 (PECy7) (BD Biosciences 561314), and 1:20 IgM-peridinin-chlorophyll-protein (PerCP)-Cy5.5 (BD Biosciences 561285). Sorted IgA^+^ B cells were activated as previously described (47). Briefly, cells were plated 2 cells/well into 384-well plates containing 10,000 irradiated 3T3-CD40L (provided by Nicole-Doria Rose, Mascola Laboratory, Vaccine Research Center, NIH) cells per well in a medium containing 100 U/mL interleukin (IL)-2 (Roche 11147528001) and 50 ng/mL IL-21 (Thermo Fisher Scientific, PHC0211). Cells were cultured for 2 weeks following which supernatants were harvested and screened by high-throughput flow cytometry. The supernatants were either mixed with streptavidin beads (Spherotech SVFA-2552-6K, SVFB-2552-6K, SVFA-2558-6K, SVFB-2558-6K) coated with biotinylated full-length recombinant PfCSP protein or PfCSP peptides (see table S2 for sequence information) and incubated for 30 minutes at 4˚C, or with *P*. *falciparum* NF54 sporozoites labeled with 1:2000 SYBR Green, and incubated for 1 hour at 4˚C. Without washing, the beads were incubated for 30 minutes at 4˚C with either 1 μg/mL goat anti-human IgA-Dylight 405 (Jackson Immunoresearch 109-475-011) or 1 μg/mL goat anti-human IgA-Alexa Fluor 647 (Jackson Immunoresearch 109-606-011) and read with the iQue Screener Plus high-throughput flow cytometer. FACS data were analysed on the Forecyt program (Intellicyt).

### Monoclonal antibody sequence analysis and production

cDNA was synthesized from B cell clones of interest and antibody heavy and light chains were PCR-amplified using gene-specific primers (41) and sequenced as previously described (48). Sequence analysis including the determination of heavy chain variable (V_H_) and light chain variable (V_L_) genes and percentage of somatic mutations was done using the International Immunogenetics Information System (IMGT) database (49). Plasmids containing the V_H_ with IgA1 or IgA2 constant region and V_L_ genes were synthesized (Genscript) and used to transfect Expi293 (Thermo Fisher Scientific) cells without a J chain to express only monomeric IgA. Recombinant IgA was purified by fast performance liquid chromatography using a Tricon column (GE Healthcare) containing the CaptureSelect IgA Affinity Matrix (Thermo Fisher Scientific). The column was attached to an AKTA chromatographic system (GE Healthcare). After loading of the culture media, the column was washed with endotoxin-free PBS. The bound IgA was then eluted with 0.1 M Glycine-HCl, pH 2.7 and the eluates were immediately neutralized with 1 M Tris-HCl, pH 8. The IgA preparations were then dialyzed extensively against PBS for complete buffer exchange. The antibodies were also expressed with an IgG1 backbone and purified using HiTrap Protein A columns (GE Healthcare). For select antibodies for which Fabs were expressed, heavy chain plasmids encoding only the V_H_ and C_H_1 were synthesized and used to transfect Expi293 cells along with light chain plasmids. Fab purification was done with CaptureSelect KappaXP Affinity Matrix (Thermo Fisher Scientific).

### Detection of *P*. *falciparum* infection by PCR

As previously described (3), we adapted a nested PCR technique (50) to amplify parasite DNA directly from DBS using primers targeting the human *Plasmodium* species 18S ribosomal RNA gene. *Plasmodium* positive samples were identified only as *P*. *falciparum*, *P. malariae*, or both (mixed infections) given the negligible incidence of other *Plasmodium* species in the study area. Target bands were detected with a DNA 5 K LabChip on the LabChip GX HT (Caliper Lifesciences) per the manufacturer’s protocol.

### VRC 314 samples

Plasma and peripheral blood mononuclear cell (PBMC) samples were obtained from healthy malaria-naive adults (18-45 years of age) enrolled in the VRC 314 clinical trial (https://clinicaltrials.gov/; trial number NCT02015091) after obtaining written informed consent. VRC 314 was a phase 1, open-label, dose-escalation trial that was designed to assess the safety, immunogenicity, and protective efficacy of the Sanaria PfSPZ Vaccine (radiation-attenuated, aseptic, purified, cryopreserved *Plasmodium falciparum* NF54 sporozoites) (51). Samples in this study were obtained from two groups of individuals; one group was immunized with 3 doses of 900,000 PfSPZ Vaccine, followed by controlled human malaria infection (CHMI) with 5 infectious mosquito bites (*P*. *falciparum* 3D7), and the other group underwent CHMI without prior immunization.

### Plasma binding to *P*. *falciparum* sporozoites and infected erythrocytes

*P*. *falciparum* NF54 sporozoites were dissected from the salivary glands of *Anopheles stephensi* and harvested in PBS containing 10 μM E-64 protease inhibitor (Sigma-Aldrich E3132). 5000 sporozoites were stained with 1:1500 SYBR Green and 1:30 Malian or U.S. plasma for 30 minutes at 4˚C, washed, and then incubated with 1:105 mouse anti-human IgM-BV450 (BD Biosciences 561286), 2.5 μg/mL goat anti-human IgA-Alexa Fluor 594 (Jackson Immunoresearch 109-586-011), and 2.5 μg/mL goat anti-human IgG-Alexa Fluor 647 (Jackson Immunoresearch 109-606-170) for 30 minutes at 4˚C. A mixture of 4 strains of *P*. *falciparum* infected erythrocytes was used for the screen with Malian plasma; the laboratory line 3D7, a Cambodian isolate CP806, and two Malian isolates (Mali G and Mali K). The blood-stage parasites were cryopreserved in glycerolyte at the late trophozoite stage and thawed with a series of salts according to standard procedure (52). A mixture of 0.5 million cells (including uninfected erythrocytes) from each culture was stained with 16 nM Hoechst 33342 (Thermo Fisher Scientific 62249) and 1:30 Malian plasma for 30 minutes at 4˚C, washed and then incubated with 2.5 μg/mL goat anti-human IgM-Alexa Fluor 488 (Jackson Immunoresearch 109-546-129), 2.5 μg/mL goat anti-human IgA-Alexa Fluor 594 (Jackson Immunoresearch 109-586-011), and 2.5 μg/mL goat anti-human IgG-Alexa Fluor 647 (Jackson Immunoresearch 109-606-170) for 30 minutes at 4˚C. The binding data with sporozoites and infected erythrocytes were acquired with the BD Fortessa X-20 (BD Immunocytometry Systems) and analyzed by FlowJo software (Tree Star). Binding to sporozoites was calculated based on the median fluorescence intensity (MFI) of each fluorophore, and binding to infected erythrocytes was measured based on the percentage of late-stage parasites stained by plasma due to the antigenic heterogeneity on the infected erythrocyte surface.

For the comparison of sporozoite-specific IgA, IgG, and IgM in Malian individuals before, during and after an acute febrile malaria episode, 2000 sporozoites were stained with 1:50 plasma as above, followed by the addition of one of three secondary antibodies: 2.5 μg/mL goat anti-human IgG-Alexa Fluor 647 (Jackson Immunoresearch 109-606-170), 2.5 μg/mL goat anti-human IgA-Alexa Fluor 647 (Jackson Immunoresearch 109-606-011), or 2.5 μg/mL goat anti-human IgM-Alexa Fluor 647 (Jackson Immunoresearch 109-606-129). The samples were analyzed with the iQue Screener (Intellicyt).

### Plasma and monoclonal antibody binding to PfCSP

Streptavidin beads with different intensities of PE-channel fluorescence (Spherotech SVFA-2558-6K and SVFB-2558-6K) were incubated with the following biotinylated antigens: 10 μg/mL recombinant PfCSP (aa 21-375 from PlasmoDB ID: PF3D7_0304600.1), 5 μg/mL N-terminus (aa 21-104 from PlasmoDB ID: PF3D7_0304600.1), 5 μg/mL C-terminus (aa 273-375 from PlasmoDB ID: PF3D7_0304600.1), 2 μg/mL NANP (NANPNANPNANPNANPNANPNANPNANPNANPNANP), or 10 μg/mL CD4 (53) or TV1-gp140 as a negative control antigen for 30 minutes at room temperature. The beads were blocked with 10 μg/mL of CD4 for 30 minutes at room temperature, washed and mixed. For the Malian plasma, the beads were stained with 1:20 plasma for 30 minutes at room temperature, washed, and split into 3 for staining with 2.5 μg/mL goat anti-human IgA Alexa Fluor 647 (Jackson Immunoresearch 109-606-011), goat anti-human IgG Alexa Fluor 647 or goat anti-human IgM Alexa Fluor 647 (Jackson Immunoresearch 109-606-129). For the VRC 314 plasma, the beads were stained with four dilutions of plasma for 30 minutes at 4˚C (1:20, 1:100, 1:500, and 1:/2500), washed, and incubated with 2.5 μg/mL goat anti-human IgA Alexa Fluor 647 for 30 minutes at 4˚C. For the IgA monoclonal antibodies, the beads were stained with 12 dilutions of each antibody, washed, and incubated with 2.5 μg/mL goat anti-human IgA-Alexa Fluor 647 (Jackson Immunoresearch 109-606-011) as described above. The beads were read with the iQue Screener Plus (Intellicyt) high-throughput flow cytometer and data were analyzed with FlowJo software (Tree Star). Data from titrations were analyzed by calculating area under the curve (AUC) for the titration and dividing by pre-immunization AUC (VRC 314 data) or AUC for the negative control antigen (monoclonal antibody data) to obtain MFI ratios. Malian plasma data were normalized by dividing MFI by the negative control antigen MFI for each plasma.

### Memory B cell staining from VRC 314 trial

Cryopreserved PBMCs from the VRC 314 trial were thawed and stained for 30 minutes at room temperature in the dark with the following panel: 1:500 Blue LIVE/DEAD (Thermo Fisher Scientific L23105), 18 μg/mL IgA-Dylight 405 (Jackson Immunoresearch 109-475-011), 1:50 CD14-BV510 (BioLegend 301842), 1:50 CD3-BV510 (BioLegend 317332), 1:100 CD8-BV510 (301048), 1:50 CD56-BV510 (BioLegend 318340), 1:50 CD27-BV605 (BioLegend 302830), 1:40 CD21-BV711 (BD Biosciences 563163), 1:50 CD19-BV750 (BioLegend 302236), 1:125 IgD-PECy7 (BD Biosciences 561314), 1:20 IgM-Brilliant Ultraviolet (BUV)395 (BD Biosciences 563903), 1:125 CD38-Alexa Fluor 700 (BD Biosciences 560676), 1:40 IgG-allophycocyanin (APC)H7 (BD Biosciences 561297), 1:25 PfCSP-Brilliant Blue (BB)660, and 1:25 PfCSP-BUV737. The PfCSP probes were made by conjugating biotinylated PfCSP to streptavidin tagged with the relevant fluorescent dye. The cells were sorted using a BD FACS Aria II and analyzed with FlowJo software (Tree Star). Cells were gated on live CD19^+^CD14^-^CD3^-^CD8^-^CD56^-^CD21^+^CD27^+^IgA^+^/IgG^+^, with CD27^++^CD38^++^ cells excluded.

### In vivo liver burden reduction assay in mice

As previously described (41), female 6-to 8-weeks old B6(Cg)-Tyrc-2J/J albino mice (Jackson Laboratories) were intravenously injected with 300 μg of test monoclonal antibodies diluted in a total volume of 200 μL PBS and challenged intravenously with 2,000 chimeric *P*. *berghei* sporozoites expressing PfCSP (Pb-PfCSP) within 2 hours. Two days post-challenge, the mice were administered intraperitoneally 150 μl of 30 mg/mL Rediject D-luciferin (Perkin Elmer) and imaged by the IVIS Spectrum in vivo imaging system (Perkin Elmer) to quantify the liver-stage parasite burden. Total flux (photons/second) was measured after choosing an equivalent region of interest around each mouse liver. Liver-stage parasite burden was compared to negative control mice that did not receive a monoclonal antibody and naïve mice that did not receive sporozoites. All mouse research was conducted according to National Institutes of Health (NIH) guidelines and approved by the Vaccine Research Center animal care and use ethics committee (Animal Study Protocol VRC-17-702).

### Plasma binding from immunized and mosquito-bitten malaria-naïve individuals to sporozoites

*P*. *falciparum* NF54 sporozoites dissected from salivary glands were stained with 1:2000 SYBR Green and four dilutions of VRC 314 U.S. plasma (1:20, 1:100, 1:500, and 1:2500) for 30 minutes at 4˚C, washed, and then incubated with 2.5 μg/mL goat anti-human IgA-Alexa Fluor 647 (Jackson Immunoresearch 109-606-011) or goat anti-human IgG-Alexa Fluor 647 (Jackson Immunoresearch 109-606-170) for 30 minutes at 4˚C. The binding data with the sporozoites were acquired with the iQue Screener Plus high-throughput flow cytometer and analyzed with FlowJo software (Tree Star).

### Mouse intradermal challenge of Pb-PfCSP sporozoites

Four-to six-week-old female BALB/c mice were purchased from Jackson Laboratories and challenged by a single intradermal injection on the left ear with 3-3.6 × 10^4^ chimeric Pb-PfCSP sporozoites. Control mice were injected with a pool of matched salivary grand extract from uninfected mosquitoes. Six days later, cells were isolated from left (ipsilateral) and right (contralateral) auricular lymph nodes, portal and celiac lymph nodes, Peyer’s patches, mesenteric lymph nodes, and spleen for flow cytometry. Live cells were pre-incubated for 10 minutes at 4˚C with 1:100 anti-CD16/CD32 mAb to block Fcγ receptors II/III and 1:1000 LIVE/DEAD Fixable aqua (Thermo Fisher Scientific L34966), and then stained for 15 minutes at 4˚C with 1:250 anti-mouse CD19-APCeF780 (Invitrogen 47-0193-82), 1:250 GL7-Alexa Fluor 647 (BD Biosciences 561529), 1:250 CD95-BV421 (BD Biosciences 562633), 1:100 CD138-BV786 (BD Biosciences 740880), 1:100 IgA-PE (Invitrogen 12-4204-82), 1:100 IgG1-BUV805 (BD Biosciences 741942), 1:100 IgG2a-BUV805 (BD Biosciences 749396), 1:100 IgG2b-BUV805 (BD Biosciences 749136), 1:100 IgG3-BUV805 (BD Biosciences 749007), 1:100 NANP tetramer-FITC, and 1:100 NANP tetramer -BV605 in BD brilliant stain buffer (BD Biosciences 563794), and fixed with BD Cytofix/Cytoperm (BD Biosciences 51-2090KZ) per manufacturer’s instructions. The NANP probes were made by conjugating biotinylated NANP to streptavidin tagged with the relevant fluorescent dye. After fixation, intracellular IgA and IgG were further stained with IgA-PE, IgG1-BUV805, IgG2a-BUV805, IgG2b-BUV805, IgG3-BUV805, NANP tetramer-FITC, and NANP tetramer-BV605 in BD brilliant stain buffer as above. Data were acquired using an BD LSRFortessa X-20 flow cytometer with FACSDiva software (BD Biosciences) and analyzed using FlowJo software (Tree Star). All mouse research was conducted according to National Institutes of Health (NIH) guidelines and approved by the Vaccine Research Center (VRC) animal care and use ethics committee (Animal Study Protocol VRC-17-702).

### Titration of IgA and IgG monoclonal antibodies on *P*. *falciparum* sporozoites

*P*. *falciparum* NF54 sporozoites dissected from salivary glands or midguts were stained with 1:2000 SYBR Green and dilutions of IgA or IgG monoclonal antibodies for 30 minutes at 4˚C, washed, and then incubated with 2.5 μg/mL goat anti-human kappa-Alexa Fluor 647 (Southern Biotech 2062-31) or 2.5 μg/mL goat anti-human lambda-Alexa Fluor 647 (Southern Biotech 2072-31) for 30 minutes at 4˚C. To compare the binding of MAD2-6 IgA to live and dead sporozoites, the sporozoites were stained with a range of dilutions of the antibody for 30 minutes at 4˚C, followed by staining with 2.5 μg/mL IgA-Alexa Fluor 647 (Jackson Immunoresearch 109-606-011) for 30 minutes at 4˚C. Sporozoites were labeled with 1 μg/mL mAb10-Dylight 405 and stained with 5 μg/mL of propidium iodide prior to loading onto the flow cytometer. Data were acquired with the iQue Screener Plus high-throughput flow cytometer and analyzed with FlowJo software (Tree Star).

### Heparin and MAD2-6 binding and competition ELISA

96-well half-area plates were coated with 100 μg/mL heparin or MAD2-6 IgA, incubated at 37°C for 1 hour, and blocked with 1% BSA in PBS. Serially diluted P14-P19 PfCSP peptides and recombinant PfCSP (see table S2 for sequence information) were added to the plate and incubated for 1 hour at room temperature. Next, 1:500 goat anti-human IgG Fc-alkaline phosphatase (Southern Biotech 2048-04) was added to the plate for a 1 hour incubation, followed by p-nitrophenyl phosphate (p-NPP) substrate (Sigma). The plates were read with the EnSpire plate reader (Perkin Elmer) at 405 nm after 1 hour.

In competition experiments, plates were coated with 100 μg/mL MAD2-6 IgA or IgG overnight at 4°C and blocked with 1% BSA in PBS. A mixture of heparin (Fresenius Kabi 401586J) at a range of concentrations and 0.5 μg/mL of biotinylated P17 was added to the plates. Next, streptavidin-alkaline phosphatase (Southern Biotech 7100-04) was added to the plate, and the plates were developed and read after 30 minutes as described above.

### Multiplex plate-based fluorescence assay (Uplex)

Mapping of MAD2-6, 5D5, and BL18 mAbs was performed using PfCSP overlapping peptides (peptides 13-22) that were 15 amino acids in length (GenScript) and overlapped by 11 residues spanning the N-terminal region of PfCSP. The assay was performed using the MSD U-Plex Assay platform (Meso Scale Discovery) according to the manufacturer’s instructions.

### Biolayer interferometry

Kinetics of antibody binding to PfCSP peptides were measured using streptavidin-capture biosensors in the Octet Red384 system (fortéBio). Assays were performed with agitation at 30°C. Monoclonal antibodies were plated in solid black tilt-well 384-well plates (Geiger Bio-One). Loading of the biotinylated P17 peptide was done for 300 seconds, followed by immersing the biosensors into buffer (PBS + 1% BSA) for 60 seconds to assess baseline assay drift. Association with whole IgG (serially diluted from 2 μM to 31 nM) was performed for 300 seconds, followed by dissociation in buffer for 600 seconds. Data analysis and curve fitting were performed using Octet Data Analysis software. Experimental data were fitted with the binding equations describing a 1:1 heterologous ligand interaction. Global analyses of the complete data sets, assuming binding was reversible (full dissociation), were carried out using nonlinear least-squares fitting allowing a single set of binding parameters to be obtained simultaneously for all concentrations of a given monoclonal antibody dilution series.

### Structural biology

MAD2-6 IgG Fab was concentrated to 10 mg/mL and mixed with the EKLRKPKHKKLKQ peptide, which overlaps with P17 (KLRKPKHKKLKQPAD) recognized by MAD2-6 mAb in the initial screening, in a 5:1 molar ratio of peptide to Fab. Upon identifying the minimal epitope of MAD2-6 IgG Fab, MAD2-6 IgA Fab was crystallized with a shorter peptide, KPKHKKLKQ, at the same concentration and molar ratio. The peptides used for co-crystallization were ordered from Innopep Inc. with a purity of >98% and contained chlorine counter ions. Crystal screening was carried out using a high-throughput, robotic CrystalMation system (Rigaku) at The Scripps Research Institute, which was based on the sitting drop vapor diffusion method with 35 μL reservoir solution and each drop consisting of 0.1 μL protein + 0.1 μL precipitant. High-quality crystals of the MAD2-6 IgG Fab-peptide co-complex diffracted to 2.14 Å and were grown in a reservoir solution containing 2.4 M ammonium sulfate and 0.1 M MES pH 6.0. Crystals of MAD2-6 IgA Fab-peptide co-complex diffracted to 2.50 Å and were grown in a well solution containing 20% polyethylene glycol 3350 and 0.2 M potassium citrate pH 8.3. Crystals for both co-complexes were grown at 20˚C and appeared after 3 days. MAD2-6 IgG and IgA Fab co-complex crystals were cryoprotected by soaking in a well solution supplemented with 20% glycerol and 20% ethylene glycol, respectively, before being flash-cooled in liquid nitrogen. X-ray diffraction data were collected at the Advanced Proton Source (APS) beamline 23ID-B. The datasets were indexed, integrated, and scaled using the HKL-2000 package (54). The structures were determined by molecular replacement (MR) using Phaser (55), with a crystal structure of Fab311 (ref. 18, PDB 6AXK) as a search model for MAD2-6 IgG Fab. The MAD2-6 IgA Fab structure was solved using the variable domain from the MAD2-6 IgG Fab and the constant domain from PDB: 3M8O (30) as the MR search models. Structure refinement was performed using phenix.refine (56) and iterations of refinement using Coot (57). Amino-acid residues of the Fabs were numbered using the Kabat system, and the structures were validated using MolProbity (58). Hydrogen bonds were assessed with the program HBPLUS (59). The crystal structures have been deposited in the PDB database with PDB ID: 7K75, and 7K76 for MAD2-6 IgA and IgG Fabs, respectively.

### Statistical Analysis

Associations between plasma IgA binding to sporozoites, plasma IgG binding to sporozoites, plasma IgA binding to PfCSP and donor age were evaluated using a two-tailed Pearson’s correlation. Segmented regression of plasma IgA versus age was performed using the “segmented” package library in R and the median age was used as the initial value of the breakpoint for the two segments. The association between P17-specific IgA and IgG (n = 525 subjects) and the number of febrile malaria episodes during the ensuing transmission season was determined using age-adjusted Poisson regression analysis, with Bonferroni correction. In the in vivo experiment comparing the efficacy of N-terminus antibodies, differences in parasite liver burden between each antibody and the no mAb control were determined using the Kruskal-Wallis with Dunn’s multiple comparison test for panels with multiple antibodies and the two-tailed Mann-Whitney test for the panel with MAD2-302 and MAD3-23. Total fluorescence intensity of CSP-stained sporozoites and trails between the MAD2-6, 2A10 and buffer control were compared using one-way ANOVA with Dunnett’s multiple comparisons. In the figure legends, N refers to the number of independent experiments unless stated otherwise.

## Supplementary Material

Supplementary Materials

Data File S1

## Data Availability

All data associated with this study are in the paper or supplementary materials. The sequences of the antibody heavy and light chains have been deposited in NCBI GenBank under accession numbers MZ076644-MZ076659. Crystal structures have been deposited in the PDB database with PDB ID: 7K75 and 7K76 for MAD2-6 IgA and IgG Fabs, respectively. The IgA monoclonal antibodies are covered by a materials transfer agreement (NIAID/NIH). Correspondence and requests for materials should be addressed to J.T. and P.D.C.

## One Sentence Summary

A functional human IgA response to malaria targets a conserved epitope in the N-terminus of the *Plasmodium falciparum* circumsporozoite protein.
