# Supplementary material for: Functional human IgA targets a conserved site on malaria sporozoites
Source: Sci Transl Med. Author manuscript; Available in PMC 2021 Jul 12. (PMC7611206; doi:10.1126/scitranslmed.abg2344)
Supplement: Supplementary Materials [file EMS127541-supplement-Supplementary_Materials.docx]

Supplementary Materials for

Functional human IgA targets a conserved site on malaria sporozoites

Joshua Tan^1†*^, Hyeseon Cho^2†^, Tossapol Pholcharee^3†^, Lais S. Pereira^4^, Safiatou Doumbo^5^, Didier Doumtabe^5^, Barbara J. Flynn^4^, Arne Schön^6^, Sachie Kanatani^7^, Samantha O. Aylor^8^, David Oyen^3^, Rachel Vistein^4^, Lawrence Wang^4^, Marlon Dillon^4^, Jeff Skinner^2^, Mary Peterson^2^, Shanping Li^2^, Azza H. Idris^4,9^, Alvaro Molina-Cruz^10^, Ming Zhao^11^, Lisa Renee Olano^11^, Patricia J. Lee^8^, Alison Roth^8^, Photini Sinnis^7^, Carolina Barillas-Mury^10^, Kassoum Kayentao^5^, Aissata Ongoiba^5^, Joseph R. Francica^4^, Boubacar Traore^5^, Ian A. Wilson^3,12#^, Robert A. Seder^4#^, Peter D. Crompton^2#*^

Correspondence to: tanj4@nih.gov and pcrompton@niaid.nih.gov

**This PDF file includes:**

Materials and Methods

Fig. S1. Antibody responses to sporozoites in naturally infected individuals.

Fig. S2. Antibody responses to sporozoites in malaria-naïve individuals.

Fig. S3. NANP-specific IgA plasmablast response in mice after intradermal inoculation of PbPfCSP sporozoites.

Fig. S4. Effect of isotype on binding to sporozoites and PfCSP.

Fig. S5. Effects of deglycosylating IgA monoclonal antibodies.

Fig. S6. Binding of MAD2-6 to PfCSP and peptide 17.

Fig. S7. In vitro binding and functional assays with MAD2-6.

Fig. S8. Structural basis for recognition of the N-terminal epitope containing region I by MAD2-6 IgG Fab.

Fig. S9. Individual residue contributions to the buried surface area (BSA) of Fab-Fab interface.

Table S1. Isotype and variable, diversity and joining (VDJ) gene usage of the IgA monoclonal antibodies.

Table S2. Sequences of full-length PfCSP and PfCSP peptides.

Table S3. X-ray data collection and refinement statistics.

**MATERIALS AND METHODS**

**Memory B cell FluoroSpot** **assay**

Peripheral blood mononuclear cells (PBMCs) from vaccinated subjects were stimulated with 2.5 µg/mL of CpG ODN-2006 (Invivogen tlrl-2006), protein A from *Staphylococcus aureus* Cowan strain at a 1:10,000 dilution (Sigma-Aldrich P7155), or 0.5 µg/mL of pokeweed mitogen (Sigma-Aldrich L8777) for 5 days in Iscove's Modified Dulbecco's Medium (IMDM) medium (Thermo Fisher Scientific 31980-030) containing 10% heat-inactivated fetal bovine serum (FBS) and MycoZap (Lonza VZA-2021). Human immunoglobulin (Ig)G/IgA/IgM FluoroSpot kit (MABTECH FS-050617-10) was used to detect IgA- or IgG-secreting B cells as instructed by the manufacturer. For the detection of *Plasmodium falciparum* circumsporozoite protein (PfCSP)-specific IgA and IgG, the plate was coated with recombinant PfCSP protein at a final concentration of 5 µg/mL. Spots were read and quantified by ZellNet Consulting, Inc.

**Malian memory B cell (MBC) screening**

CD19^+^IgM^-^IgD^-^ IgG^+^ and IgA^+^ MBCs were sorted from PBMCs as described in the main Materials and Methods. MBCs were incubated for 6 days in Memory Activation Medium (Berkeley Lights) in 96-well U-bottom plates (Corning) at 2,500 cells/well (100 µL/well). On day 6, 15 µL of culture supernatants were harvested and incubated with 15 µL of 10 µg/mL PfCSP- or 10 µg/mL Pf merozoite surface protein-1 (MSP1)-labeled beads (*53*) for 30 minutes at room temperature, stained with 2.5 µg/mL goat anti-human IgG-Alexa Fluor 647 (Jackson Immunoresearch 109-606-170) or goat anti-human IgA Alexa Fluor 647 (Jackson Immunoresearch 109-606-011) for 30 minutes at room temperature, and analyzed on the iQue Screener (Intellicyt).

**Mouse time course experiment**

Female 6- to 8-week old B6(Cg)-Tyrc-2J/J albino mice (Jackson Laboratories) were intravenously injected with 200 µg of glycosylated MAD2-6 IgA, tailpiece-deglycosylated IgA, or IgG diluted in a total volume of 200 µL phosphate buffered saline (PBS). Serum samples were taken at 30 minutes, 2 hours, 6 hours, 10 hours, and 24 hours. 96-well half-area plates (Corning) were coated with 10 µg/mL of unlabeled anti-human IgA (Southern Biotech 2050-01) or anti-human IgG (Southern Biotech 2040-01) overnight at 4ºC. The plates were blocked with 1% bovine serum albumin (BSA) in PBS for 1 hour at room temperature and incubated with dilutions of the serum samples for 1 hour at room temperature. Next, the plates were incubated with 1:500 goat anti-human IgA-alkaline phosphatase (Southern Biotech 2050-04) or goat anti-human IgG-alkaline phosphatase (Southern Biotech 2050-04) for 1 hour at room temperature, followed by incubation with substrate (para-nitrophenyl phosphate (p-NPP), Sigma). The plates were read at 405 nm using an Enspire MultiMode Plate Reader (PerkinElmer).

**Deglycosylation of IgA monoclonal antibodies**

IgA monoclonal antibodies were incubated with 1 unit of PNGaseF (NEB P0704L) /µg of IgA for 4 hours at 37ºC. After PNGase F treatment, the enzyme was separated from the deglycosylated monoclonal antibodies by size-exclusion chromatography with a Superose 6 Increase 10/300 GL column (GE Healthcare) that was pre-equilibrated with PBS. The column was run at 0.5 mL/min and fractions were collected as 0.5 mL/tube. The major antibody fractions were pooled for further applications.

**Mass spectrometry**

Approximately 3 µg of each antibody sample was reduced with 5 mM dithiothreitol (DTT) at 56˚C for 1 hour. The sample was then diluted 5X with 100 mM ammonium bicarbonate to reduce the DTT concentration. 300 ng of sequencing grade trypsin (V5111, Promega) was added to the IgA sample and incubated overnight at 37˚C. The digested sample was then dried with a speedvac and resuspended in 12 µl liquid chromatography-mass spectrometry (LC-MS) sample buffer (0.1% Formic acid, 3% acetonitrile). The samples were stored at 4˚C before LC-MS/MS analysis.

Acquisitions were performed on an Orbitrap Fusion Lumos mass spectrometer (Thermo Fisher Scientific) equipped with an EASY-Spray Ion Source and an Easy-nLC 1200. Liquid chromatography was performed using a PepMap C18 trap column (3µm, 75µm ID, 3 cm) and an EASY-Spray PepMap RSLC C18 analytical column (2µm, 75µm ID, 25 cm) operating at a 500 nL/minute flow rate with mobile phases A (water; 0.1% formic acid) and B (80% acetonitrile; 20% water (v/v); 0.1% formic acid). The 100 minute acquisition cycle was comprised of an 80 minute gradient from mobile phase A to 50% mobile phase B followed by a rapid 5 minute ramp to 100% mobile phase B where it was held for 5 minute before the column was re-equilibrated to 100% A. Acquisitions were performed in data-dependent mode with a cycle time of 3 seconds with a full MS scan of all ions from m/z 200 to mz/ 2000 at a resolution of 120,000 (m/z 190 at the target value of 4E5). Precursor ions with charges from 2-8 at an intensity threshold of 2E4 were fragmented using CID and detected in the OrbiTrap at 30,000 resolution using an isolation window of 1.6 m/z a q of 0.25 and a collision energy of 35%. Dynamic exclusion was enabled with the duration of 10 seconds.

Data files were processed using the suite of Preview, Byonic, and Byologic (v3.6; Protein Metrics Inc). Preview analysis did not show unexpected amounts of sample handling modifications. Byonic searches were performed against a concatenated database containing the sequences of interest and the human proteome from the Uniprot KB/Swiss-Prot (11/2019) using a semi-specific tryptic digestion with a 3 parts per million (ppm) precursor tolerance, a 100 ppm fragment tolerance and 2 missed cleavages. Common and rare modifications were capped at 1 with oxidation of methionine and deamidation of asparagine listed as common and the Protein Metrics’ 309 mammalian no sodium N-glycan database used for the rare glycan search. Protein output was capped at a 1% false discovery rate (FDR) using a decoy database approach. Database matches associated with the heavy and light chain sequences of interest were processed and quantified using Byologic with quantitation of deamination enabled.

**SDS-PAGE**

2.5 µg of each antibody was loaded onto a 4-12% NuPAGE gradient gel (Thermo Fisher Scientific) and run at 120V for 1-2 hours. The gel was stained with PageBlue Staining Solution (Thermo Fisher Scientific) and destained with multiple rinses of water.

**Conservation of MAD2-6 IgA binding site in global isolates**

PfCSP (ID: Pf3D7_0304600) sequences from >2000 global *P. falciparum* isolates were obtained using the Panoptes Pf3k program (<https://www.malariagen.net/apps/pf3k/release_3/index.html>) and viewed on a genome browser (IGV, Broad Institute) to identify indel and substitution mutations in the binding site of MAD2-6 IgA covering P16 and P17.

**P17 peptide mutagenesis enzyme-linked immunosorbent assay (ELISA)**

MA6000 384 SA microtiter plates (Meso Scale Discovery) were blocked with PBS + 5% BSA (20 μL/well). Blocked plates were coated with 10 μL/well of biotinylated PfCSP Peptide 17 variants where each residue was mutated to an alanine or serine if the original reside was an alanine (0.1 μg/mL, Genscript) in PBS + 1% BSA for 1 hour at room temperature. The coated plates were incubated for 1 hour at room temperature with 10 μL of mAb MAD2-6 IgA at varying concentrations (0.41 – 300.0 μg/mL, 3-fold serial dilutions). Plates were then incubated with 10 μl/well of 1.0 mg/mL Sulfo-tag goat anti–human IgA (Meso Scale Discovery) for 1 hour at room temperature. Plates were washed five times with PBS-Tween between each step. After a final wash, 35 μL of 1X MSD Read T Buffer (Meso Scale Discovery) was added to each well and plates were analyzed on an MSD Sector Image 600 instrument.

**P17 variant competition ELISA**

Competition ELISA was performed using the unlabeled PfCSP Peptide 17 variants described above. Briefly, ELISA plates were coated with 10 μl of PfCSP Peptide 17 (0.1 μg/mL). After coating, MAD2-6 mAb (5 μg/mL) preincubated overnight with varying concentrations (0–2,000 μg/mL) of PfCSP Peptide 17 variants in PBS with 1% BSA were added to the coated plates, and ELISA was performed on the MSD platform as described above.

**Malian plasma and P17 ELISA**

96-well half-area plates were coated with 1 µg/mL P17 overnight at 4ºC and blocked with 1% BSA in PBS. Malian plasma at a 1:100 dilution was added to the plates, followed by 1:500 goat anti-human IgG-alkaline phosphatase (Southern Biotech 2040-04) or goat anti-human IgA-alkaline phosphatase (Southern Biotech 2050-04) (both for 1 hour incubations). The plates were developed and read after 1 hour as described above.

**Isothermal titration calorimetry (ITC)**

All ITC experiments were performed at 25°C using a VP-ITC from MicroCal/Malvern Instruments. Binding experiments with full-length CSP (FL-CSP) were performed by injecting either IgA or IgG at a concentration of ~50 µM (expressed per antigen binding site) into the stirred calorimetric cell containing FL-CSP at a concentration of about 0.3 µM. Experiments with peptide 17 were performed in two different ways: i) antibody at a concentration of 50 - 60 µM antigen binding sites was injected into the cell containing the peptide at 2 µM; ii) peptide at a concentration of 66 µM was injected into the calorimetric cell containing antibody at a concentration of 3 µM antigen binding sites. The reagents were prepared in PBS, pH 7.4. The injection volume was kept constant at 7 or 10 µL for each complete experiment. The exact concentrations of FL-CSP and the antibodies were determined from the absorbance at 280 nm. The concentration of peptide 17 was determined by a total nitrogen assay (*60*). The heat evolved upon each injection was obtained from the integral of the calorimetric signal and the heat associated with binding was obtained after subtraction of the heat of dilution. The enthalpy change, ΔH, the association constant, Ka (the dissociation constant, Kd =1/Ka) and the stoichiometry, N, were obtained by nonlinear regression of the data to a single-site binding model. Gibbs energy, ΔG, was calculated from the binding affinity using ΔG = -RTlnKa, (R = 1.987 cal/(K × mol)) and T is the absolute temperature in kelvin). The entropy contribution to Gibbs energy, -TΔS, was calculated from the relation ΔG = ΔH -TΔS. All results are expressed per antigen binding site and the stoichiometry, N, denotes the number of antigen binding sites per mole of either FL-CSP or peptide 17.

**CSP reaction**

Freshly dissected *P. falciparum* salivary gland NF54 sporozoites in Hank´s Balanced Salt Solution with 2% bovine serum albumin (HBSS/BSA) at pH 7.4 were mixed with the antibody solution to be tested. Sporozoites with antibody were placed on a microscope slide, covered with a coverslip and sealed with nail polish. The slide was placed in a petri dish with wet kimwipes and incubated for 30 minutes at 37°C. 100 sporozoites per condition were scored for the CSP reaction using phase-contrast microscopy (Nikon E600).

**Trail gliding assay**

15,000 freshly dissected *P. falciparum* salivary gland NF54 sporozoites in 50 µl of HBSS/BSA pH 7.4 were mixed 1:1 with the antibody solution in HBSS. The mixture was pre-incubated for 30 minutes at 20°C and then transferred to a 96 well glass bottom plate (Greiner, 655892) coated with 5 µg/mL of mAb 2A10 which is specific for the PfCSP repeat region in PBS. The plate was centrifuged for 3 minutes at 300 × g and incubated for 1 hour at 37°C. Wells were fixed in 4% paraformaldehyde in PBS, blocked with 1% BSA in PBS (pH 7.4) and stained with biotinylated mAb 2A10 in 1% BSA in PBS (pH 7.4) for 1 hour at room temperature, followed by detection with Alexa Fluor 488 streptavidin (Invitrogen) diluted at 1:500 in PBS for 1 hour at room temperature. Samples were preserved in a glycerol / PBS solution (ratio, 9:1) at 4°C and imaging was performed on 25 positions per well (5 x 5, 500 µm apart) by using ImageXpress Micro XLS Widefield high-content analysis system (Molecular Devices) with 40X Plan fluor objective. Acquired images were processed using Fiji (https://fiji.sc/) to measure total fluorescence intensity.

***P. falciparum* mosquito infections for inhibition assay**

*P. falciparum* (strain NF54)-infected mosquitoes were obtained from WRAIR Entomology and were maintained according to standard methods previously described (*61, 62*). When stage V gametocytes were prevalent (day 14−17 of gametocyte cultures) the culture was used to infect day 4−6 old mosquitoes. Infections of laboratory-reared *Anopheles stephensi* (Nijmegen strain) were accomplished using a water-jacketed glass feeder system and kept at 80% humidity in 26 ºC environmental chambers with 10% sucrose supplemented water.

**Infection of 384-well plate with *P. falciparum* sporozoites and Inhibition of Liver-Stage Development Assay (ILSDA).**

The *P. falciparum* liver stage experiments were performed as previously described (*63, 64*). Briefly, cryopreserved primary human hepatocytes (PHH) (Cat No. M00995-P, donor YNS and BGW, BioIVT, Inc.) were thawed following manufacturer’s protocol and diluted in hepatocyte culture medium (HCM) (InVitroGroTM CP Medium, BioIVT, Inc.) to a concentration of 900 cells/μL (18,000 PHHs/well). The hepatocytes were seeded as a confluent monolayer in a commercial 384-well plate (Cat No. 781956, Greiner) and at 2 days post seed each well received a complete media change. Salivary glands were dissected at 17 days post-infected blood feed from infected *A. stephensi* mosquitoes and collected into 100 μL of dissection media, followed by gland disruption. Sporozoites were counted using a hemocytometer and diluted accordingly (1,000 sporozoites/μL) in HCM. The anti-NANP repeat mAb 2A10, MRA-183A, was obtained through Biodefense and Emerging Infections Research (BEI) Resources, NIAID, NIH (Hybridoma 2A10 Anti-*Plasmodium falciparum* Circumsporozoite Protein, contributed by Elizabeth Nardin), and used as the assay positive control. The test protein (MAD2-6 IgA) and positive control were diluted in HCM and tested at the final concentrations of 200, 50, 12.5 and 2.5 ng/μL. Collected sporozoites were added to conditioned wells in quadruplets and exposed for 24 hours at 37°C before washing with HCM. Media was changed every 2 days using HCM with 1% penicillin-streptomycin-neomycin until fixation 5 days post infection for immunofluorescence assays.

At 5 days post infection, wells were fixed with 4% paraformaldehyde for 20 minutes at room temperature and washed thrice with 1x PBS. The experimental wells were incubated in blocking buffer (1% BSA and 0.3% Triton X in 1x PBS) with mouse anti-GAPDH conjugated to AlexaFluor-555 (1:1,000-fold dilution), obtained from The European Malaria Reagent Repository (http://www.malariaresearch.eu), overnight at 4°C. After, the wells were washed three times with 1X PBS and incubated for 1 hour at room temperature with Hoechst 33342 (10 µg/mL at 1:2,000-fold dilution), then washed three times and filled with 1X PBS for imaging and storage.

Parasite growth was determined by quantification of developing liver stage schizonts based on fluorescent intensity and morphology then analyzed using an Operetta CLS Imaging System and Harmony software 4.16 (Perkin Elmer). Percent inhibition was calculated using the infected control wells and the following equation, % inhibition = 100 x (1 – (X/(Average of Infected Controls)), where X is the average number of parasites per test condition. Statistical significance of parasite growth was determined using one-way ANOVA followed by Dunnett’s multiple comparisons correction to the infected control where values are represented by *P <* 0.0001 (****) and no significance (ns) as defined in Prism (GraphPad). Images of the *P. falciparum* liver stage schizont were captured with a water immersion 20x objective on an Operetta CLS Imaging System (Perkin Elmer). The image z-stacks were processed, and a scale bar added with ImageJ (version 2.1.0/1.53c).

**Isolation of BL18**

Rhesus macaques were immunized with full-length recombinant PfCSP formulated in polyinosinic-polycytidylic acid, and poly-L-lysine (poly-ICLC) adjuvant (Hiltonol, Oncovir, Inc.) at weeks 0, 4, and 22 of the study. PBMCs were harvested at week 24 from Ficoll purification of blood drawn from the femoral artery, as previously described (*65*). The PBMCs were stained for 45 minutes at 4ºC with 1:40 CD20-Alexa Fluor 700 (made in-house), 1:25 IgM-PeCy5 (BD Biosciences 551079), 1:10 IgG-APC (BD Biosciences 550931), 1:50 CD3-APC-Cy7 (BD Biosciences 557757), 1:100 CD14-BV785 (BioLegend 301839), 1:200 CD8-BV450 (BD Biosciences 560347), along with P16-17 tetramer-FITC and PfCSP N-terminus tetramer-PE to sort for antigen-specific cells. Probe-positive B cells were single-cell sorted into 96-well PCR plates for amplification of antibody heavy and light chains, as previously described (*41*).


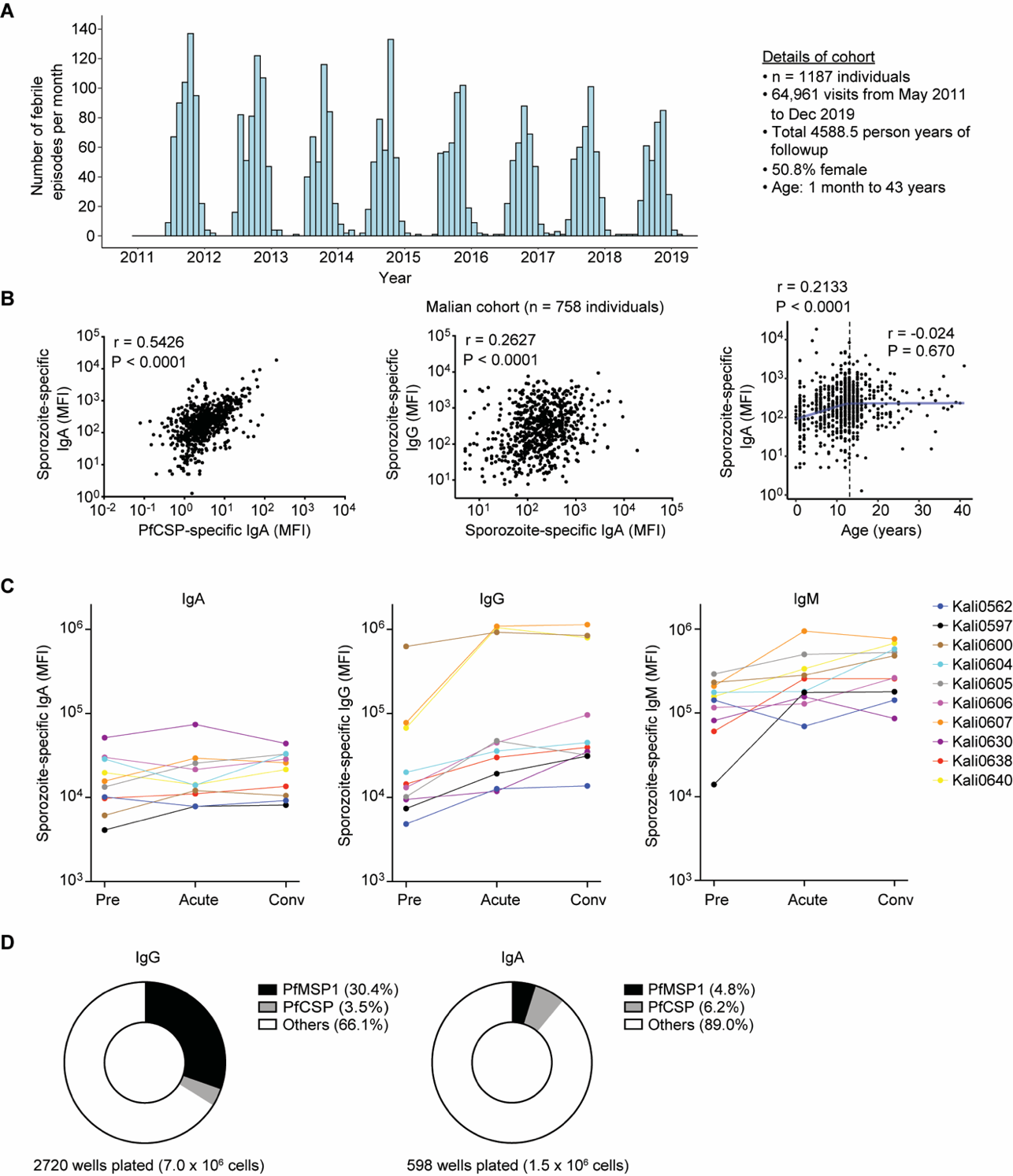


**Fig. S1. Antibody responses to sporozoites in naturally infected individuals. (A)** Frequency of clinical malaria episodes per month in the Kalifabougou, Mali cohort from 2011 to 2020. Clinical malaria defined as axillary temperature ≥37.5°C, ≥2500 asexual parasites/μL of blood, and no other cause of fever discernible on physical examination. **(B)** Spearman correlation between sporozoite-specific IgA and PfCSP-specific IgA; sporozoite-specific IgA and IgG; and sporozoite-specific IgA and age in the Malian cohort. PfCSP values were normalized to a negative control antigen. MFI, median fluorescence intensity. For sporozoite-specific IgA versus age (right panel), a segmented regression analysis was performed on the data, followed by separate Spearman correlation analysis of data points before and after the breakpoint. **(C)** Sporozoite-specific IgA, IgG and IgM in Malian individuals before the 2016 malaria season (Pre), at the date of confirmed blood-stage malaria (Acute) and one week after the confirmed diagnosis (Conv). **(D)** Frequency of IgA^+^ and IgG^+^ memory B cells (MBCs) targeting PfMSP1 and PfCSP. The B cells were plated at 2500 cells per well; percentages refer to the number of positive wells.

**Fig. S2. Antibody responses to sporozoites in malaria-naïve individuals. (A)** Binding of plasma IgG, IgM and IgA to *P. falciparum* sporozoites among 43 malaria-naïve individuals in the U.S. MFI, median fluorescence intensity. **(B)** PfCSP- and peptide-specific plasma IgA responses in U.S. volunteers after immunization with 3 x 900,000 cryopreserved, irradiated sporozoites (PfSPZ Vaccine) (N = 2 independent experiments). Red arrows indicate time points of controlled human malaria infection (CHMI). MFIs were normalized to pre-immunization values. **(C)** PfCSP-specific IgA^+^ and IgG^+^ MBCs in a second sporozoite-immunized donor (608). Gated on live CD19^+^CD14^-^CD3^-^CD8^-^CD56^-^CD21^+^CD27^+^IgA^+^/IgG^+^ cells, with CD27^++^CD38^++^ plasmablasts excluded. BUV, Brilliant Ultraviolet; BB, Brilliant Blue. **(D)** Frequency of PfCSP-specific IgA^+^ (green, upper) and IgG^+^ (red, lower) B cells by enzyme-linked immunospot (ELISpot) in donors 602 and 611 pre-immunization and at week 30 (14 weeks after the third immunization). **(E)** Sporozoite-specific plasma IgG responses in U.S. volunteers after immunization with 3 x 900,000 cryopreserved, irradiated sporozoites (Sanaria PfSPZ Vaccine) (N = 2 independent experiments). MFIs were normalized to pre-immunization values. **(F)** Sporozoite-specific plasma IgG responses in malaria-naïve U.S. volunteers after CHMI with 5 *P. falciparum*-infected mosquitos (N = 2 independent experiments). MFIs were normalized to pre-immunization values.

**Fig. S3. NANP-specific IgA plasmablast response in mice after intradermal inoculation of PbPfCSP sporozoites.** Mice were injected with 36,000 sporozoites in the left ear and cells were analyzed 6 days after injection (n = 3 mice per group, N = 2 independent experiments). Gated on live CD138^+^IgA^+^ cells. FITC, fluorescein; BV, Brilliant Violet; PBs, plasmablasts; LN, lymph node.

**Fig. S4. Effect of isotype on binding to sporozoites and PfCSP. (A)** Binding of MAD2-6 IgA to live and dead *P. falciparum* sporozoites. In the right panel, the colored dots show a titration of MAD2-6 IgA from 83 µg/mL to 0.3 µg/mL. The black dots show binding of a control non-specific mAb at 83 µg/mL. mAb10-Dy405 was added just before flow cytometry to identify sporozoites. FSC-A, forward scatter area; FSC-H, forward scatter height; Dy, Dylight. **(B)** Titration of MAD2-6 IgA on live and dead sporozoites. MFI, median fluorescence intensity. **(C)** Sporozoite binding of IgA versus IgG monoclonal antibodies from MAD3 (originally isolated as IgA) (N = 2 independent experiments). Antibody binding was detected using the same secondary anti-light chain antibody to allow comparison between the isotypes. **(D)** Ratio of IgA to IgG binding of monoclonal antibodies to PfCSP, NANP and P17 (N = 2 independent experiments). Antibody binding was detected using the same secondary anti-light chain antibody to allow comparison between the isotypes. Only ratios for binding to full-length PfCSP and the major PfCSP epitope of each antibody are shown. AUC, area under the curve.

**Fig. S5. Effects of deglycosylating IgA monoclonal antibodies. (A)** Time course experiment showing decay of fully glycosylated MAD2-6 IgA, PNGaseF-deglycosylated IgA, and IgG in C57BL/6 mice (n = 3 mice per group). Each mouse was injected intravenously with 200 µg of antibody and followed up to 24 hours post injection. Horizontal bars show the mean. **(B)** Molecular weights of fully-glycosylated MAD2-6 IgA, native deglycosylated MAD2-6 IgA and PNGaseF-deglycosylated MAD2-6 IgA on an SDS-PAGE gel. Both non-reducing and reducing lanes are shown. The native deglycosylated IgA refers to MAD2-6 IgA that lost sugars at the tailpiece glycan site after storage at 4ºC for several weeks. **(C)** Mass spectrometry data showing glycosylation states of N267 (constant region) and N463 (tailpiece) in fully-glycosylated MAD2-6 IgA (gly), native deglycosylated MAD2-6 IgA (degly native) and PNGaseF-deglycosylated MAD2-6 IgA (degly PNGaseF). Treatment with the PNGaseF enzyme only affected the tailpiece glycan but left N267 largely unaffected. **(D)** Efficacy of glycosylated IgA versus PNGaseF-deglycosylated IgA in the in vivo Pb-PfCSP challenge model (n = 5 mice per group) where total flux in photons per second (p/s) indicates liver parasite burden. Horizontal bars show geometric mean.

**Fig. S6. Binding of MAD2-6 to PfCSP and peptide 17. (A)** Alanine scan of P17 with 4 extra C-terminal residues (sequence: KLRKPKHKKLKQPADGNPD) (N = 2 independent experiments). Each position shows % binding of MAD2-6 IgA to a peptide carrying a single alanine substitution relative to the wild-type (WT) peptide. **(B)** Binding of MAD2-6 IgG to P17 with competition from heparin, as measured by ELISA (N = 1). The dotted line shows the baseline signal with no peptide added. **(C)** Isothermal titration calorimetry (ITC) measurements of MAD2-6 IgA binding to the P17 peptide and PfCSP (N = 2 independent experiments). The high value of the stoichiometry of ~12 for binding to PfCSP obtained from the nonlinear regression of the data was ignored because of the error associated with ITC data for low-affinity binding events. Q, heat energy; K_d_, dissociation constant; ΔH, enthalpy change; N, stoichiometry. **(D)** Isothermal titration calorimetry showing MAD2-6 IgG binding to P17 and PfCSP (N = 2 independent experiments). The high value of the stoichiometry of ~9 for binding to PfCSP obtained from the nonlinear regression of the data was ignored because of the error associated with ITC data for low-affinity binding events.

**Fig. S7. In vitro binding and functional assays with MAD2-6. (A)** Binding of MAD2-6 IgG to midgut (MG) and salivary gland (SG) sporozoites (N = 1). MFI, median fluorescence intensity. **(B)** Binding of a control anti-NANP repeat antibody, mAb10 IgG, to MG and SG sporozoites (N = 2 independent experiments). **(C)** Representative images of *P. falciparum* NF54 sporozoites post incubation at indicated concentration of MAD2-6 IgA, mAb 2A10, and no antibody control. mAb 2A10 targets the NANP repeat region of PfCSP and was used as a positive control for the CSP reaction. Scale bars, 5 µm. **(D)** The percentage of *P. falciparum* NF54 sporozoites displaying the CSP reaction post incubation at indicated concentration of MAD2-6 IgA, mAb 2A10, and no antibody control. 100 sporozoites were scored for each condition. **(E)** Quantification of *P. falciparum* NF54 sporozoites with (motile) and without trails (non-motile) post incubation at indicated concentration of MAD2-6 IgA, mAb 2A10, and no antibody control. Images were acquired from 25 positions per condition and CSP-stained sporozoites were assessed (n = 594-702 sporozoites per condition). **(F)** The total fluorescence intensity of CSP-stained *P. falciparum* NF54 sporozoites and trails. 25 images per condition were analyzed and no significant differences were observed between MAD2-6 IgA and the no antibody control (One-way ANOVA, Dunnett’s multiple comparisons, Control versus 100 µg/mL P = 0.2366, Control versus 200 µg/mL P = 0.6920, Control versus 2A10 P < 0.0001). Red bars indicate the mean total fluorescence intensity. **(G)** Representative images of CSP-stained *P. falciparum* NF54 sporozoites and trails at indicated concentrations of MAD2-6 IgA and mAb 2A10. Scale bars, 50 µm. **(H)** Inhibition of *P. falciparum* NF54 liver stage development by MAD2-6 IgA. mAb 2A10 was used as a positive control. Graph bars represent means with standard deviation (s.d.) of quadruplicate data (N = 1 experiment). Statistical significance was determined using one-way ANOVA followed by Dunnett’s multiple comparisons to the infected control. At all mAb 2A10 concentrations, P < 0.0001. **(I)** Representative image of liver-stage *P. falciparum* NF54 parasite from infected control well. Blue, Hoechst; orange, GAPDH (liver-stage parasite). Scale bar, 5 µm.


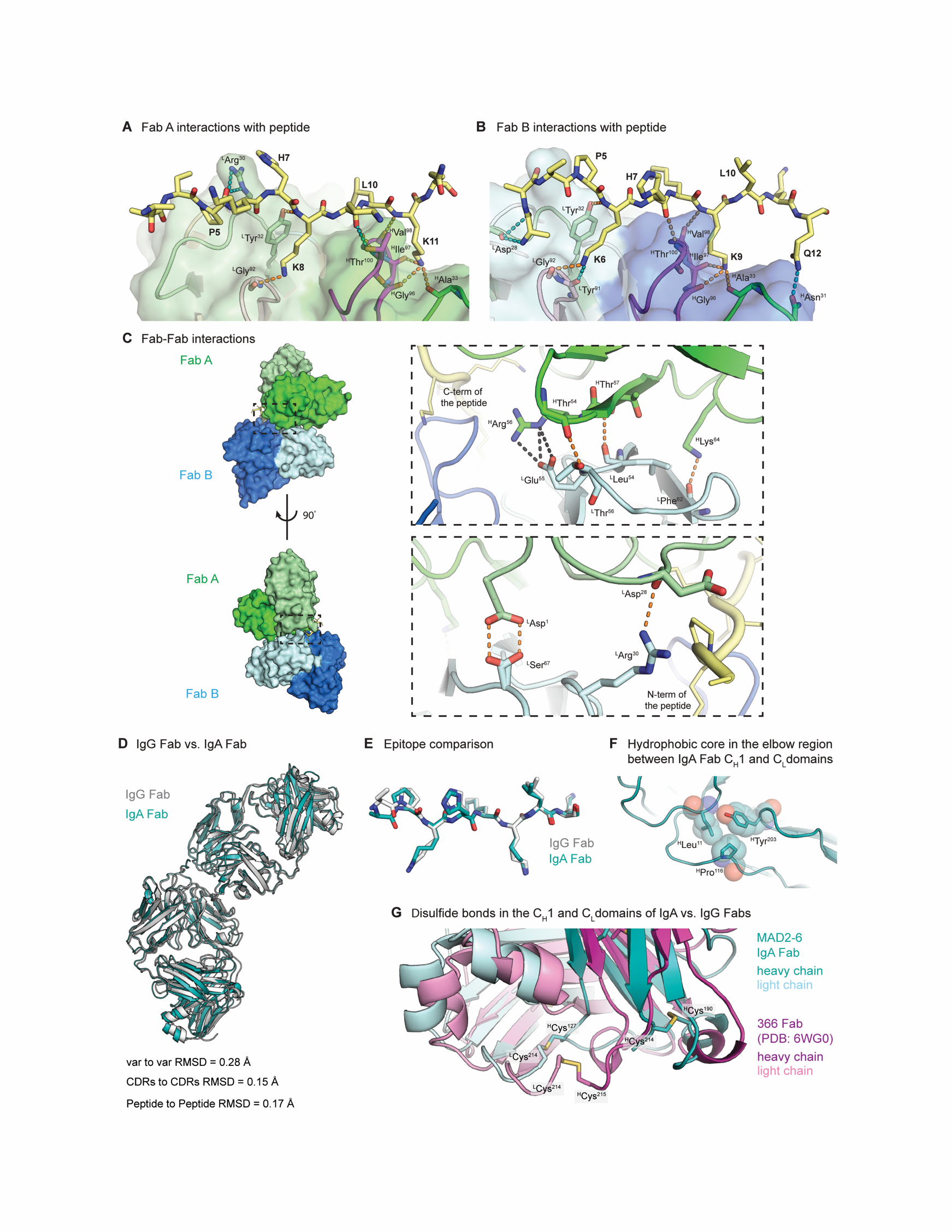


**Fig. S8. Structural basis for recognition of the N-terminal epitope containing region I by MAD2-6 IgG Fab. (A and B)** Interaction of MAD2-6 IgG Fab A (A) and Fab B (B) with the P17 peptide. Fabs are displayed in ribbon representation overlayed with transparent surfaces (Fab A: light green and green for light and heavy chain; Fab B: light blue and blue for light and heavy chain). Complementarity-determining regions (CDRs) are colored light green, light cyan, pink, magenta, and green for CDR L1, L2, L3, H3, and H1, respectively. Hydrogen bonds (dashed lines) that are shared in both Fab A and B are shown in orange, whereas those unique to each Fab are shown in teal. The peptide is represented as yellow sticks. Fab residue numbers are shown with superscript H and L for heavy and light chain, respectively. **(C)** Homotypic interactions of two MAD2-6 IgG Fabs. The Fab variable domains are shown as surfaces (Fab A: light green and green represent light and heavy chain; Fab B: light blue and blue represent light and heavy chain) and ribbon representation in the enlarged box with the same coloring scheme for heavy and light chains (not for CDRs) as for the surfaces. The peptide is displayed in ribbon representation with side chains as sticks. Black and orange dashes represent salt bridges and hydrogen bonds, respectively. **(D)** Comparison of MAD2-6 IgA and IgG Fab structures. Alignment of MAD2-6 IgA (blue) and IgG (grey) using the peptides for alignment. The root mean square deviation (RMSD) of the alignment using the variable domains (var), CDRs, and the peptides are shown. **(E)** Close-up view of the alignment of the peptides from MAD2-6 IgA (blue) and IgG (grey) Fabs. Only the overlapping Lys4-Lys11 residues are shown. **(F)** The hydrophobic core in the elbow region of the heavy chain of MAD2-6 IgA Fab. The heavy chain is shown in teal ribbons with the side chain of residues in the hydrophobic core shown as sticks overlaying with sphere representation. **(G)** Comparison of disulfide bonds in the constant domains of IgA and IgG Fabs. The Fabs are displayed in ribbons with different colors for the heavy and light chains indicated. The cysteine side chains forming a disulfide bond are shown in gold sticks.

**
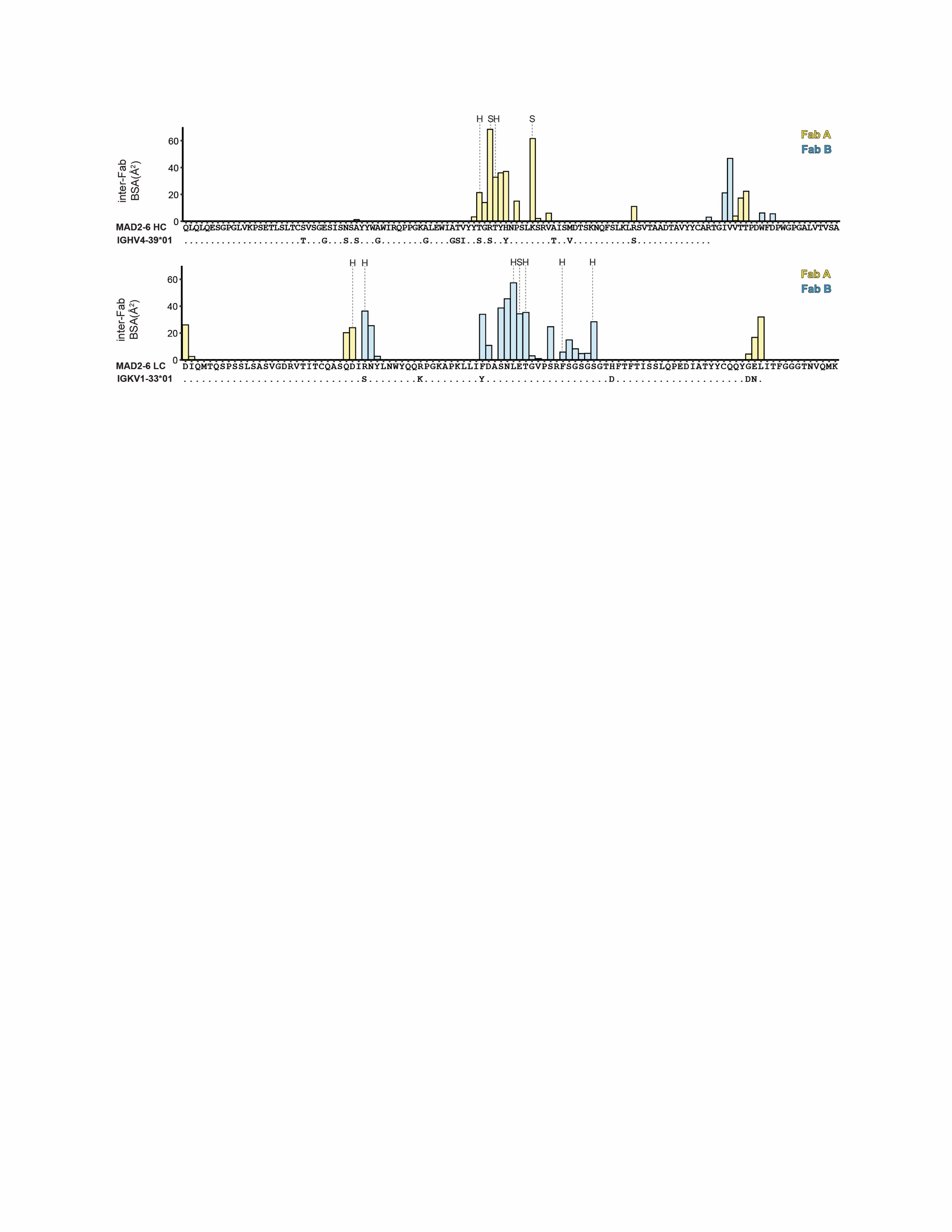
**

**Fig. S9. Individual residue contributions to the buried surface area (BSA) of Fab-Fab interface.** The BSA of inter-Fab interactions are shown in a bar plot for the heavy and light chains of MAD2-6 IgG Fab. The yellow and blue bars indicate the BSA on Fab A and Fab B (defined as in Fig. 4), respectively. The alignment between the Fab heavy and light chain sequences and germline immunoglobulin heavy chain variable (IGHV) and immunoglobulin kappa chain variable (IGKV) gene sequences are shown to display somatically mutated residues. “H” and “S” mark residues that are engaged in hydrogen bonds and salt bridges, respectively.

**Table S1. Isotype and variable, diversity and joining (VDJ) gene usage of the IgA monoclonal antibodies.** H, heavy; L, light.

| **Donor** | **mAb** | **Isotype** | **Light** | **VH gene** | **% VH** | **DH gene** | **JH gene** | **%JH** | **VL gene** | **%VL** | **JL gene** | **%JL** |
| --- | --- | --- | --- | --- | --- | --- | --- | --- | --- | --- | --- | --- |
| MAD2 | MAD2-6 | IgA1 | κ | VH4-39 | 89.4 | DH5-12 | JH5 | 86.3 | VK1-33 or VK1D-33 | 95.3 | JK4 | 89.2 |
|  | MAD2-60 | IgA1 | κ | VH3-33 | 96.9 | DH1-26 | JH4 | 89.6 | VK3D-15 | 97.9 | JK4 | 97.4 |
|  | MAD2-257 | IgA1 | κ | VH3-15 | 93.9 | DH2-21 | JH4 | 79.2 | VK2-30 | 97.6 | JK1 | 100 |
|  | MAD2-302 | IgA1 | κ | VH3-33 | 91.7 | DH2-15 | JH5 | 88.2 | VK1-12 or VK1D-12 | 94.6 | JK2 | 97.2 |
| MAD3 | MAD3-14 | IgA1 | λ | VH4-39 | 94.9 | DH3-9 | JH3 | 92.0 | VL9-49 | 97.6 | JL1 | 100 |
|  | MAD3-23 | IgA1 | κ | VH3-30 or VH3-30-5 | 93.7 | DH3-16 | JH4 | 89.6 | VK1D-12 | 92.1 | JK4 | 94.7 |
|  | MAD3-65 | IgA1 | κ | VH3-23 or VH3-23D | 93.1 | DH4-17 | JH3 | 92.0 | VK1-39 or VK1D-39 | 96.1 | JK1 | 94.7 |
|  | MAD3-83 | IgA2 | κ | VH1-2 | 98.3 | DH3-22 | JH4 | 93.8 | VK2-28 or VK2D-28 | 99.3 | JK2 | 97.4 |

Table S2. Sequences of full-length PfCSP and PfCSP peptides. See Fig. 2B for relative positions of each peptide. P13-P16 are N-terminal to P17, and P18-P22 are C-terminal to P17. P13-P22 are shifted 4 amino acids relative to each other.

| Protein/peptide | Sequence |
| --- | --- |
| PfCSP | QEYQCYGSSSNTRVLNELNYDNAGTNLYNELEMNYYGKQENWYSLKKNSRSLGENDDGNNEDNEKLRKPKHKKLKQPADGNPDPNANPNVDPNANPNVDPNANPNVDPNANPNANPNANPNANPNANPNANPNANPNANPNANPNANPNANPNANPNANPNANPNANPNANPNANPNVDPNANPNANPNANPNANPNANPNANPNANPNANPNANPNANPNANPNANPNANPNANPNANPNANPNANPNANPNKNNQGNGQGHNMPNDPNRNVDENANANSAVKNNNNEEPSDKHIKEYLNKIQNSLSTEWSPCSVTCGNGIQVRIKPGSANKPKDELDYANDIEKKICKMEKCSHHHHHH |
| N-term | QEYQCYGSSSNTRVLNELNYDNAGTNLYNELEMNYYGKQENWYSLKKNSRSLGENDDGNNEDNEKLRKPKHKKLKQPADGNPDP |
| NANP | NANPNANPNANPNANPNANPNANPNANPNANPNANP |
| C-term | NKNNQGNGQGHNMPNDPNRNVDENANANSAVKNNNNEEPSDKHIKEYLNKIQNSLSTEWSPCSVTCGNGIQVRIKPGSANKPKDELDYANDIEKKICKMEKCS |
| P13 | SRSLGENDDGNNEDN |
| P14 | GENDDGNNEDNEKLR |
| P15 | DGNNEDNEKLRKPKH |
| P16 | EDNEKLRKPKHKKLK |
| P17 | KLRKPKHKKLKQPAD |
| P18 | PKHKKLKQPADGNPD |
| P19 | KLKQPADGNPDPNAN |
| P20 | PADGNPDPNANPNVD |
| P21 | NPDPNANPNVDPNAN |
| P22 | NANPNVDPNANPNVD |

**Table S3. X-ray data collection and refinement statistics.**

| **Data collection** | **MAD2-6 IgA Fab** | **MAD2-6 IgG Fab** |
| --- | --- | --- |
| Beamline | 23-IDB | 23-IDB |
| Wavelength (Å) | 1.03317 | 1.03316 |
| Space group | P2_1_2_1_2_1_ | P2_1_2_1_2_1_ |
| Unit cell parameters (Å, °) | a=73.14, b=153.68, c=193.09 | a=69.39, b=77.32, c=181.15 |
|  | α=β=γ=90 | α=β=γ=90 |
| Resolution (Å) | 50.00-2.50 (2.54-2.50)^a^ | 77.32-2.14 (2.20-2.14)^a^ |
| Unique Reflections | 75,359 (3,252) | 54,785 (4,429)^a^ |
| Redundancy | 5.0 (2.9)^a^ | 6.6 (6.8)^a^ |
| Completeness (%) | 99.0 (87.1)^a^ | 100.0 (100.0)^a^ |
| <I/σ_I_> | 10.3 (0.8)^a^ | 10.0 (3.5)^a^ |
| R_sym_^b^ (%) | 12.3 (78.9)^a^ | 11.9 (52.2)^a^ |
| R_pim_^b^ (%) | 6.0 (49.4)^a^ | 5.0 (21.6)^a^ |
| CC_1/2_^c^ (%) | 90.5 (55.7)^a^ | 99.6 (77.7)^a^ |
| **Refinement statistics** |  |  |
| Resolution (Å) | 41.96-2.50 | 71.11-2.14 |
| Reflections (work) | 75,186 | 54,704 |
| Reflections (test) | 3,735 | 2,000 |
| R_cryst_^d^ / R_free_^e^ (%) | 19.7/24.5 | 18.4/21.8 |
| **Number of atoms** |  |  |
| Fab | 13,105 | 6,607 |
| Peptide | 132 | 86 |
| Water | 352 | 395 |
| **Average B-value (Å^2^)** |  |  |
| Fab | 48 | 29 |
| Peptide | 42 | 37 |
| Water | 40 | 36 |
| Wilson B-value | 42 | 28 |
| **Root-mean-square deviation (RMSD) from ideal geometry** |  |  |
| Bond length (Å) | 0.008 | 0.003 |
| Bond angle (°) | 0.77 | 0.59 |
| **Ramachandran statistics^f^** |  |  |
| Favored (%) | 95.06 | 97.11 |
| Outliers (%) | 0.06 | 0.00 |
|  |  |  |

^a^ Numbers in parentheses refer to the highest resolution shell.

^b^ *R*_sym_ = Σ*_hkl_* Σ*_i_* | I*_hkl,i_* - <I*_hkl_*> | / Σ*_hkl_* Σ*_i_* I*_hkl,i_* and R*_pim_* = Σ*_hkl_* (1/(n-1))^1/2^ Σ*_i_* | I*_hkl,i_* - <I*_hkl_*> | / Σ*_hkl_* Σ*_i_* I*_hkl,i_*, where I*_hkl,i_* is the scaled intensity of the i^th^ measurement of reflection h, k, l, <I*_hkl_*> is the average intensity for that reflection, and *n* is the redundancy.

^c^ CC_1/2_ = Pearson correlation coefficient between two random half datasets.

*^d^ R*_cryst_ = Σ*_hkl_* | *F*_o_ - *F*_c_ | / Σ*_hkl_* | *F*_o_ | x 100, where *F*_o_ and *F*_c_ are the observed and calculated structure factors, respectively.

^e^ *R*_free_ was calculated as for *R*_cryst_, but on a test set comprising 5% of the data excluded from refinement.

^f^ From MolProbity^58^*.*
